# Stress and reproductive hormones reflect inter-specific social and nutritional conditions mediated by resource availability in a bear–salmon system

**DOI:** 10.1093/conphys/cou010

**Published:** 2014-05-02

**Authors:** Heather M. Bryan, Chris T. Darimont, Paul C. Paquet, Katherine E. Wynne-Edwards, Judit E. G. Smits

**Affiliations:** 1 Faculty of Veterinary Medicine, University of Calgary, Calgary, Alberta, Canada T2N 4Z6; 2 Raincoast Conservation Foundation, Sidney, British Columbia, Canada V8L 1Y2; 3 Department of Geography, University of Victoria, Victoria, British Columbia, Canada V8W 3R4

**Keywords:** Black bear, cortisol, grizzly bear, hair analysis, stable isotope analyses, testosterone

## Abstract

Food availability can influence the nutritional and social dynamics within and among species. Our investigation focused on grizzly and black bears in coastal British Columbia, Canada, where recent and dramatic declines in their primary prey (salmon) raise concerns about potentially negative effects on bear physiology. We examined how salmon availability relates to stress and reproductive hormones in coastal grizzly (*n* = 69) and black bears (*n* = 68) using cortisol and testosterone. In hair samples from genotyped individuals, we quantified salmon consumption using stable isotope analysis and hormone levels by enzyme immunoassay. To estimate the salmon biomass available to each bear, we developed a spatially explicit approach based on typical bear home-range sizes. Next, we compared the relative importance of salmon consumption and salmon availability on hormone levels in male bears using an information theoretical approach. Cortisol in grizzly bears was higher in individuals that consumed less salmon, possibly reflecting nutritional stress. In black bears, cortisol was better predicted by salmon availability than salmon consumption; specifically, individuals in areas and years with low salmon availability showed higher cortisol levels. This indicates that cortisol in black bears is more strongly influenced by the socially competitive environment mediated by salmon availability than by nutritional requirements. In both species, testosterone generally decreased with increasing salmon availability, possibly reflecting a less competitive environment when salmon were abundant. Differences between species could relate to different nutritional requirements, social densities and competitive behaviour and/or habitat use. We present a conceptual model to inform further investigations in this and other systems. Our approach, which combines data on multiple hormones with dietary and spatial information corresponding to the year of hair growth, provides a promising tool for evaluating the responses of a broad spectrum of wildlife to changes in food availability or other environmental conditions.

## Abstract (precis)

Food sources are critical to wildlife health but are being increasingly altered by humans. We found that stress and reproductive hormones in grizzly and black bears varied with salmon availability and consumption by bears but patterns differed between species. On-going salmon declines might affect bears via nutritional and social stress.

## Introduction

Resource availability can affect animal physiology via complex interactions among nutritional, ecological and social conditions. Notably, the abundance, distribution and quality of resources mediate energy gain, behaviour and social dynamics within and among species (Koenig and Borries, 2006). Moreover, nutritious resources that are clumped or easy to monopolize are expected to increase competition and can lead to rank-related differences in energy gains (Janson and van Schaik, 1988; van Schaik, 1989; Gende and Quinn, 2004). Accordingly, understanding how organisms respond physiologically to the abundance and distribution of resources could provide insight into the social and nutritional consequences of changes in food availability (Wikelski and Cooke, 2006; Hofer and East, 2012; Seebacher and Franklin, 2012; Cooke *et al.*, 2013; Dantzer *et al.*, 2013).

Stress and reproductive hormones are well suited for studies of wildlife ecophysiology. Specifically, the glucocorticoid steroid hormone, cortisol, is a general indicator of physiological responses to variation in internal or external conditions, including social challenges (i.e. allostatic load; Sapolsky *et al.*, 2000; Romero, 2004; Reeder and Kramer, 2005; Creel *et al.*, 2013). The androgen, testosterone, which may reflect male reproductive investment and activity during the breeding season (Kempenaers *et al.*, 2008), also can be modulated by social challenges, such as those that occur over access to mates or fitness-enhancing resources (Wingfield *et al.*, 2001; Oliveira, 2004). Both hormones can be measured in hair, which is thought to primarily reflect chronic hormone levels integrated over the period of hair growth (i.e. several months to years, depending on population-specific patterns of hair growth; Koren *et al.*, 2002; Macbeth *et al.*, 2010; Meyer and Novak, 2012). Steroid hormones are incorporated into hair via one or more mechanisms, including diffusion from the blood vessel that feeds the hair follicle, local synthesis of steroids by the hair follicle, and secretion from sebaceous and eccrine glands surrounding hair follicles (Pragst and Balikova, 2006; Keckeis *et al.*, 2012; Meyer and Novak, 2012). A number of recent studies have demonstrated that hormonal measurements of hair reflect biologically meaningful endocrine activity in wildlife (reviewed by Meyer and Novak, 2012), including bears (*Ursus* spp.; Macbeth *et al.*, 2010, 2012; Bourbonnais *et al.*, 2013; Bryan *et al.*, 2013b; Malcolm *et al.*, 2013).

In areas of North America where they still co-occur, coastal populations of grizzly (*Ursus arctos*) and black bears (*Ursus americanus*) have co-evolved with spawning Pacific salmon (*Oncorhynchus* spp.) as a nutritious but spatially and temporally constrained food (Jacoby *et al.*, 1999; Reimchen, 2000; Darimont *et al.*, 2010; Levi *et al.*, 2012). The fat and protein from salmon come at a critical time for bears before hibernation; pre-denning fat stores are positively correlated with the reproductive success of females the following year (Hilderbrand *et al.*, 2000; Belant *et al.*, 2006). In these systems, body size is a strong predictor of reproductive success (determined by paternity and encounters with females) in male grizzly and black bears, suggesting that eating salmon is related to male fitness in a similar manner (Kovach and Powell, 2003; Zedrosser *et al.*, 2007; Costello *et al.*, 2009).

In addition to being an important food, seasonal salmon availability influences inter- and intra-population dynamics in bears. Although typically solitary, bears cluster on salmon streams when salmon become available, leading to increased potential for direct or indirect social encounters (Egbert and Stokes, 1976; Rogers, 1987; Craighead *et al.*, 1995). Feeding aggregations often lead to establishment of social hierarchies where bears may be tolerant or extremely aggressive towards other bears, depending at least in part on availability and spatial configuration of food resources (Herrero, 1983; Rogers, 1987; Gende and Quinn, 2004). Physical encounters at food sources can lead to injury or death, especially of juveniles (Rogers, 1987; Mattson *et al.*, 1992; Mattson and Reinhart, 1995; Ben-David *et al.*, 2004). Between species, grizzly bears tend to dominate salmon spawning streams, with black bears using alternative food sources, fishing sites or times of day (Machutchon *et al.*, 1998; Belant *et al.*, 2006; Fortin *et al.*, 2007; Schwartz *et al.*, 2010). Within species there may also be a dominance hierarchy, with some individuals being excluded from prime fishing areas (Gende and Quinn, 2004; but see Gill and Helfield, 2012).

Several studies have linked food availability with cortisol in bears. Specifically, cortisol in serum is typically elevated during hibernation when bears do not eat (Harlow *et al.*, 1990; Hellgren *et al.*, 1993). Moreover, cortisol metabolites in faeces were elevated in grizzly and black bears that ate foods of low nutritional quality (Wasser *et al.*, 2004; Stetz *et al.*, 2013). In hair, higher cortisol was associated with lower salmon consumption by grizzly bears (Bryan *et al.*, 2013b) and poorer body condition in polar bears (*Ursus maritimis*; Macbeth *et al.*, 2012). In addition, human landscape disturbance also affects cortisol levels in grizzly bear hair (Bourbonnais *et al.*, 2013). In serum of male bears, testosterone rises during the breeding season (typically May–July) and remains relatively low during other parts of the year (McMillin *et al.*, 1976; Palmer *et al.*, 1988; White *et al.*, 2005). Annual patterns in testosterone are thought to be regulated largely by photoperiod; however, social conditions can also influence testosterone in bears (Garshelis and Hellgren, 1994; Bryan *et al.*, 2013b).

In coastal British Columbia (BC), Canada, widespread regional declines in salmon abundance have occurred in recent decades (Noakes *et al.*, 2000; Price *et al.*, 2008; Peterman *et al.*, 2012). Accordingly, our overarching aim was to investigate how grizzly and black bears respond physiologically to changes in salmon abundance over space and time, and how these changes relate to individual, ecological and social conditions. Based on our previous findings that cortisol decreased with increasing salmon consumption in grizzly bears (Bryan *et al.*, 2013b), we hypothesized that a negative relationship between salmon consumption and cortisol could reflect: (i) a nutritional benefit of eating salmon; or (ii) lower social stress when individuals face less competition, a context more likely when salmon are abundant relative to local bear densities. In grizzly and black bears, foraging behaviour has previously been found to follow an ideal ‘despotic’ model, in which some individuals gain and forcefully maintain access to more food than others (Beckmann and Berger, 2003; Gende and Quinn, 2004). However, the extent to which social dominance influences food intake rates also depends on resource availability (Gende and Quinn, 2004; Gill and Helfield, 2012). Therefore, we postulated that salmon availability would influence social conditions, such as the degree of competition among bears (i.e. social stress), whereas salmon consumption would reflect how much salmon a bear consumed (i.e. nutritional stress). Importantly, the relationship between salmon availability and salmon consumption is non-linear; consumption saturates at high levels of salmon abundance (Levi *et al.*, 2012). Therefore, having estimates of both salmon availability (derived from salmon biomass data) and salmon consumption by each individual bear allowed us to evaluate these hypotheses (Table 1, Hypotheses A and B).

**Table 1:** Main hypotheses tested and associated predictions examining the effects of salmon consumption and availability on cortisol and testosterone in grizzly and black bears

Hypothesis	Mechanism	Prediction	Black^a^	Grizzly^a^
(A) Cortisol is related to the amount of salmon that bears consume	Bears that consume little salmon might be nutritionally stressed	Cortisol will be negatively associated with salmon consumption^b^	Not supported	Supported
(B) Cortisol is related to social conditions mediated by salmon availability	Bears might experience lower social stress when more salmon is available	Cortisol will be negatively associated with salmon availability^c^	Supported	Not supported
(C) Testosterone is related to social conditions mediated by salmon productivity	Productive watersheds support higher bear densities, leading to increased competition for resources (such as salmon or mates) or increased investment in reproduction	Testosterone will be associated positively with watershed productivity^d^	Supported	Partly supported
(D) Testosterone is related specifically to competition for salmon	Testosterone might promote or be activated by the competitive social environment when salmon availability is low	Testosterone will be associated negatively with salmon availability^c^	Partly supported	Supported
(E) Testosterone is related to the amount of salmon that bears consume	(i) Bears with higher testosterone (e.g. older, larger or dominant individuals) might consume more salmon; and/or salmon consumption might promote reproductive activity	Testosterone will be associated positively with salmon consumption	Not supported	Not supported
	(ii) Testosterone might be lowered by contaminants in salmon; bears with lower testosterone (e.g. dominant individuals^e^) might consume more salmon; and/or both testosterone and salmon consumption might be mediated by salmon availability	Testosterone will be associated negatively with salmon consumption	Supported	Supported (Fig. 5)

^a^We considered an hypothesis to be supported if the predictor of interest was included in the top model set and showed a significant trend in the predicted direction, partly supported if the predictor was included in the top model set but was not significant, and not supported if the predictor did not occur in the top model set or was opposite to our prediction. ^b^Salmon consumption provides an estimate of a bear's yearly protein assimilation from salmon and is derived from stable isotope analysis of hair samples genetically linked to individual bears in each year. ^c^Salmon availability is a measure of the salmon biomass available to a bear in a given year relative to the average salmon biomass in that bear's home range. The metric was derived from a principal components analysis. ^d^Watershed productivity is a measure of average salmon biomass available within each bear's home range. ^e^Testosterone might be linked positively or negatively with traits such as age, body size and social rank, depending on the nature of social interactions and stability of the dominance hierarchy (Sapolsky, 1993).

We also tested a working hypothesis that testosterone would be influenced by social density, which is mediated by salmon availability among coastal bears (Hilderbrand *et al.*, 1999). Specifically, higher social density might lead to more intense competition for mates or other fitness-enhancing resources (such as salmon) and consequently, higher testosterone. This hypothesis was premised on our previous finding that testosterone was higher in coastal bears with access to salmon compared with interior bears without access to salmon (Bryan *et al.*, 2013b). Accordingly, we investigated whether testosterone varied in relationship to salmon availability within coastal populations. We specifically evaluated the relative importance of two possible mechanisms by which salmon availability might influence testosterone. Based on the established positive relationship between bear population densities and salmon availability (Hilderbrand *et al.*, 1999), we first predicted that areas with higher average salmon biomass (i.e. ‘watershed productivity’) would support more bears, possibly reflecting or promoting a more competitive social environment (Table 1, Hypothesis C). Second, we tested whether testosterone varied with salmon availability, estimated for individual bears based on year and location of capture and standardized relative to the average salmon biomass for a given watershed (i.e. ‘salmon availability’). This allowed us to investigate whether testosterone levels might be modulated specifically by competition for salmon (Table 1, Hypothesis D). In addition, we estimated the relative density of bears across regions of our study area using bears detected at hair-snagging stations. This allowed us to test whether hormone levels are influenced by variables related to bear social density, such as access to mates, independent of salmon abundance. To differentiate among these measured components of the social and ecological environments, we referred to average salmon biomass as ‘watershed productivity’, salmon biomass available to bears when sampled hair was grown as ‘salmon availability’, and density estimates from our grid-based study area as ‘bear density’. Finally, we tested hypotheses relating testosterone to salmon consumption, which could be driven by individual traits, such as age, social rank and body size, and/or by social conditions mediated by salmon availability (Table 1, Hypothesis E).

## Materials and methods

### Study area and sample collection

Our study area and sample collection protocol followed Bryan *et al.* (2013b). Briefly, we collected hair samples from black and grizzly bears on the central coast of British Columbia in May and June of 2009–2011. We divided our study area into a regular grid of square cells, within which we place barbed wire fences (i.e. hair snags) for hair collection following standard protocols (Kendall *et al.*, 2008; Proctor *et al.*, 2010). During our 2009 pilot year, we sampled from 92 5 km × 5 km grid cells over ∼2500 km^2^, and in 2010 and 2011 we sampled from 71 7 km × 7 km grid cells over ∼5000 km^2^. We checked the snags and collected hair twice in each year at intervals of 10–14 days. Snags were disassembled between consecutive years. This ∼4 week collection period occurs during the shedding phases of the annual moult. Accordingly, we assumed that hair samples represent diet and hormones assimilated in the previous year during the hair growth stage (Hilderbrand *et al.*, 1996; Schwartz *et al.*, 2003; Jones *et al.*, 2006). Additional spring hair samples from grizzly bears, obtained from the BC provincial government between 2004 and 2010, were originally collected via compulsory inspection of remains from grizzly bears hunted in coastal BC. Samples were collected under approved animal care protocols at the Universities of Santa Cruz (WILMc0904) and Calgary (BI10R-01). In addition, we obtained permission from the Heiltsuk Integrated Resource Management Department on behalf of the Heiltsuk Nation and BC Parks (Park Use Permit Number 103586).

### Hormone levels, salmon consumption and density of bears

Samples collected at hair-snagging stations were genetically linked to individual bears using seven microsatellite markers at a commercial laboratory (Wildlife Genetics International, Nelson, BC, Canada). Subsequently, we pooled samples obtained from a single individual in the same year to obtain enough material for hormone and stable isotope analyses (Table 2). As detailed in the Supplementary material, we quantified cortisol and testosterone in hair using previously validated enzyme immunoassays (Bryan *et al.*, 2013a, b). We estimated the proportion of a bear's yearly diet assimilated from salmon (i.e. its ‘salmon consumption’) using Bayesian isotope mixing models, which incorporate isotopic signatures of the samples, isotopic signatures of dietary sources (i.e. salmon and plants), fractionation of isotopes in hair, and uncertainties associated with these estimates (Supplementary material; Moore and Semmens, 2008; Semmens and Moore, 2008). Finally, within the grid-based coastal study area in which 27 of 54 grizzlies and all 59 black bears were sampled, we classified grizzly and black bear densities as high or low (Supplementary material). Density estimates were based on measures averaged over space and time and therefore could not be used directly to test associations with salmon abundance and availability.

**Table 2:** Samples used in analyses

Species	Collection method	Analyses^a^	Years	Male^b^	Female	Recapture^c^
Grizzly	Hair from government archives of hunted bears from larger coastal area	Descriptive comparison of cortisol, testosterone and diet in both sexes (Fig. 2); model selection for cortisol and testosterone in male grizzly bears	2003–2008	27	7	0
Grizzly	Hair collected from snags from 5000 km^2^ grid-based study area on central coast	Descriptive comparison of species and sexes (Fig. 2); included in both sets of models for cortisol and testosterone in grizzly bears	2008–2010	27	8	6 (5 males, 1 female)
Black	Hair collected from snags as described for grizzly bears	Descriptive comparison (Fig. 2); model selection for cortisol and testosterone in male black bears	2008–2010	59	9	9 (all male)

Hair samples were collected from grizzly and black bears from coastal British Columbia, Canada. Sample sizes represent hair from unique individuals collected in spring of a particular year. Hair samples collected from the same individual during one 4 week sampling period during the annual moult were pooled because they reflect diet and hormone levels in hair grown during the previous year. Recaptures are individuals detected in more than 1 year of the study. ^a^Results from six separate model selections are presented for male grizzly and black bears (Tables 3 and 4 and Figs 3 and 4). ^b^Only male bears were included in model selection because of the small sample size for female bears. ^c^Model sets excluding one recapture gave similar results to those including both recaptures; recaptured bears were retained in analyses.

### Salmon biomass calculations

We obtained data on numbers of spawning salmon in coastal BC from Fisheries and Oceans Canada (FOC, 2012). This database contains yearly abundance estimates of the five Pacific salmon species over 60 years in >6000 watersheds in coastal BC; however, ∼30% of species estimates from streams that are monitored regularly are missing, making comparisons over space and time inconsistent for our desired scale of analysis (Price *et al.*, 2008). Consequently, we developed an imputation method for missing data points (Supplementary material, Table S1; Ruggerone *et al.*, 2010). Validations revealed adequate agreement between imputed and existing salmon abundance estimates, with a mean coefficient of variability of 12.2% on log-transformed data (Supplementary material, Fig. S1). We then calculated salmon biomass in each watershed by multiplying abundance estimates by the average mass of salmon (in kilograms) and summing across species. We used measurements made by Groot and Margolis (1991), which are based on average mass of both sexes and assume a 1:1 sex ratio. We then divided salmon biomass by the length of the spawning area at each stream to obtain a measure of biomass per stream length for use in further calculations (BCGOV, 2006).

We estimated salmon biomass available to each bear by placing a buffer around each location where bears were detected, and calculated the spawning salmon biomass within the buffer (Supplementary material, Figs S1, S2, and S3). Given that most bears were detected only once (*n* = 50 of 59 black bears and *n* = 22 of 27 grizzly bears), we were not able to calculate home ranges individually for each bear. Consequently, we based buffer sizes for each species and sex on the largest home-range estimates calculated using the 100% minimum convex polygon method in available studies of coastal bears (Supplementary material, Fig. S3 and Table S2; Machutchon *et al.*, 1993; H. Davis, unpublished data). We generated a regular grid of 40 candidate buffers for each bear because our detection locations could have occurred anywhere within a bear's home range. We then selected the buffer containing the highest total salmon biomass, based on average biomass in the last 10 years, assuming that bears would maximize their access to this fitness-enhancing resource. Likewise, for bears detected at multiple locations within the same year, we selected the location with the highest salmon biomass. Using the buffer containing the maximum 10 year average biomass, we calculated salmon biomass in the year of hair growth and the year before hair growth. Notably, salmon availability can be affected by many factors, such as water levels, the configuration of fishing sites, the diversity of salmon species present and social interactions. For our purposes, we considered our measure of salmon abundance as the only component of availability.

### Statistical analyses

All analyses were carried out using R statistical software (R Development Core Team, 2011). Variables relating to hormone levels, salmon abundance and salmon consumption were not normally distributed, and the two species showed different degrees of skewness. Consequently, we used non-parametric Wilcoxon rank sum tests to compare the distributions of salmon consumption, cortisol and testosterone between species and sexes. To control for the number of comparisons, we adjusted *P* values using a Holm–Bonferroni correction factor.

In our subsequent model selection approach, we focused only on males, because of the small sample size for female grizzly (*n* = 14) and black bears (*n* = 9; Table 2). Our data analysis protocol generally followed that of Zuur *et al.* (2009). Initial descriptive analyses included examining variables for evidence of collinearity using pairwise correlation coefficients and variance inflation factors. Salmon biomass variables were collinear, so we used a principal components analysis on log-transformed variables to allow examination of both watershed productivity and salmon availability (Supplementary material, Fig. S4 and Table S3). The salmon consumption variable for grizzly bears was highly left skewed, with most bears eating high proportions of salmon. Accordingly, we applied an arcsine transformation of salmon consumption, which improved normality of residuals (McCune *et al.*, 2002). Cortisol and testosterone concentrations were highly right skewed, with most individuals having low concentrations of both steroids. To improve normality of residuals, a natural log transformation was adequate for black bear cortisol and testosterone. In grizzly bears, the distribution was more highly skewed, and a stronger, negative reciprocal transformation improved the normality of residuals. Four grizzly bear samples and one black bear sample had extreme values for cortisol or testosterone (>2 SD from the mean) and were not included in the analysis. An explanation for excluding these outliers and potential effects of doing so is provided by Bryan *et al.* (2013b). All variables were standardized before analysis to a mean of zero and standard deviation of one (Zuur *et al.*, 2009).

To address our specific hypotheses (Table 1), we developed sets of *a priori* candidate models describing testosterone and cortisol and grouped models into three categories (Supplementary material, Tables S4–S9). In the first group, we examined the relative importance of different environmental variables (watershed productivity, salmon availability, year and bear density). In the second group, we evaluated the most important individual variables (salmon consumption or hormone levels). Specifically, we predicted that cortisol and testosterone—both of which can influence or be affected by social interactions—should be associated positively except when nutritional stress becomes a more important modulator of cortisol than social stress. Finally, the third group of models investigated whether a combination of environmental factors, individual factors and their interactions are associated with cortisol and testosterone levels. To determine the relative importance of individual and environmental variables, we compared the top models from each group and identified associated parameters with most predictive utility.

We used multiple linear regression and Akaike's information criterion, corrected for small sample size (AICc), to rank candidate models within and among *a priori* hypotheses. The weight of evidence for individual models was calculated based on AICc scores of all models considered for each comparison. In competing top models (ΔAICc < 2) from each group, model weights were calculated relative to the top model from each group (Anderson *et al.*, 2000). We evaluated the adequacy of top models based on normality of residuals and plots of residuals vs. predicted values and residuals vs. each predictor variable (Supplementary material, Figs S5 and S6). We examined Cook's distance as an indicator of influential observations. To improve comparisons between species, we performed the analysis twice for grizzly bears, once on individual bears sampled over 8 years (*n* = 54) at a larger spatial scale and once on a subset of individuals collected between 2008 and 2010 (*n* = 27) at a smaller spatial scale (Table 2). We also performed the analyses on subsets of the data excluding individuals detected in multiple years (Table 2). Including individual as a random intercept term in the models was not possible, because only five bears were captured more than once. Randomly excluding data from one of the captures had little effect on our interpretation, so we treated bears captured in more than 1 year as independent cases in the analyses presented below.

## Results

### Salmon availability and consumption

**Figure 1:**
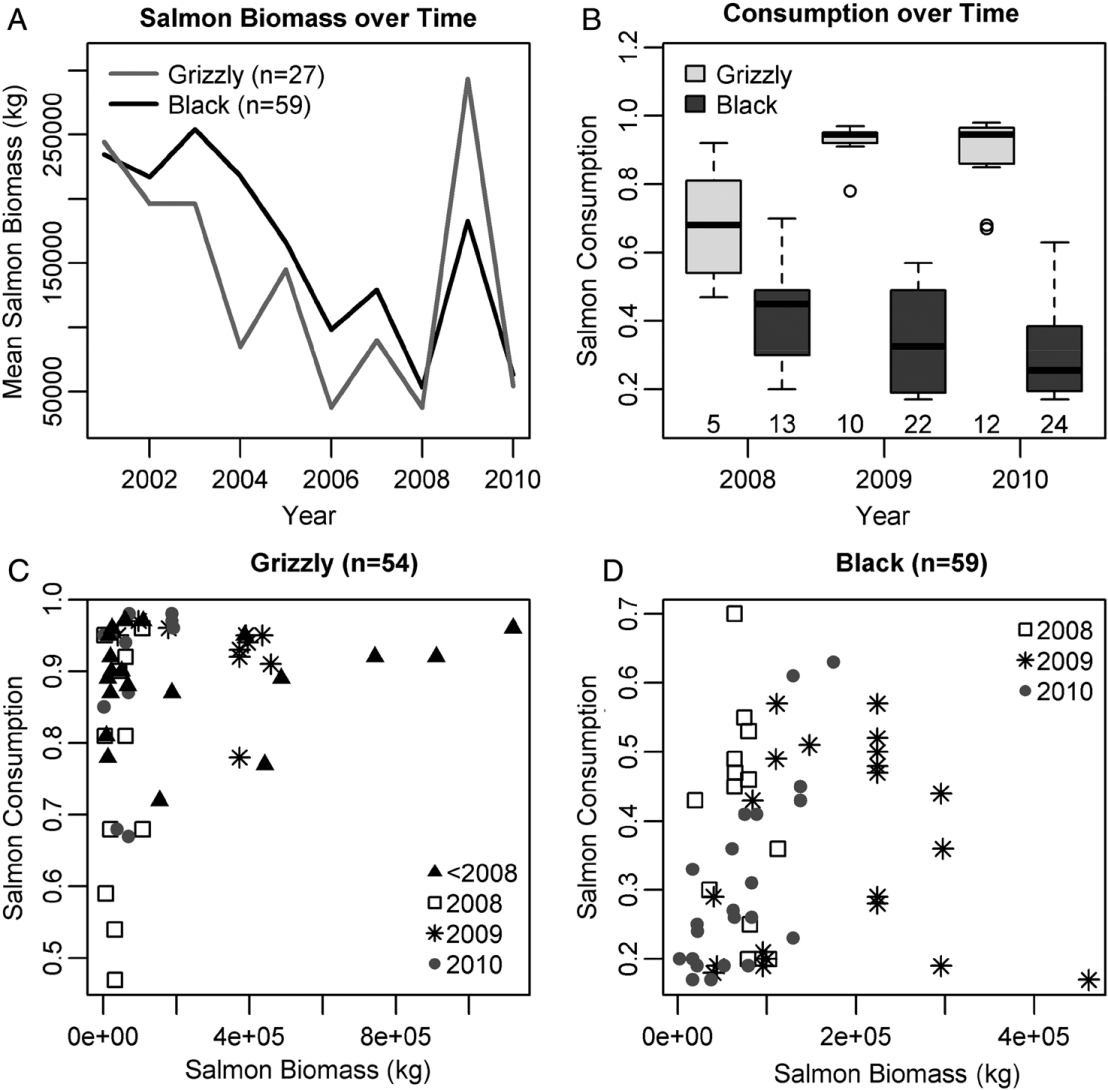
Salmon availability and consumption in male grizzly and black bears from coastal British Columbia. (**A**) Spawning salmon biomass (in kilograms) in the estimated home ranges of both species has generally decreased over the last decade, with an exception in 2009. Note that salmon biomass for grizzly and black bears is not directly comparable because different home-range size estimates were used in the two species (Table S2). (**B**) Salmon consumption (estimated from stable isotope analysis of hair) in male grizzly and black bears during the main years of the study (2008–2010). (**C** and **D**) Salmon consumption showed non-linear relationships with salmon biomass in the estimated home ranges of both grizzly (C) and black bears (D); (C) includes grizzly bears (*n* = 27) sampled from outside the core study area. Other analyses were performed on salmon consumption data for grizzly bears in Bryan *et al.* (2013b).

**Figure 2:**
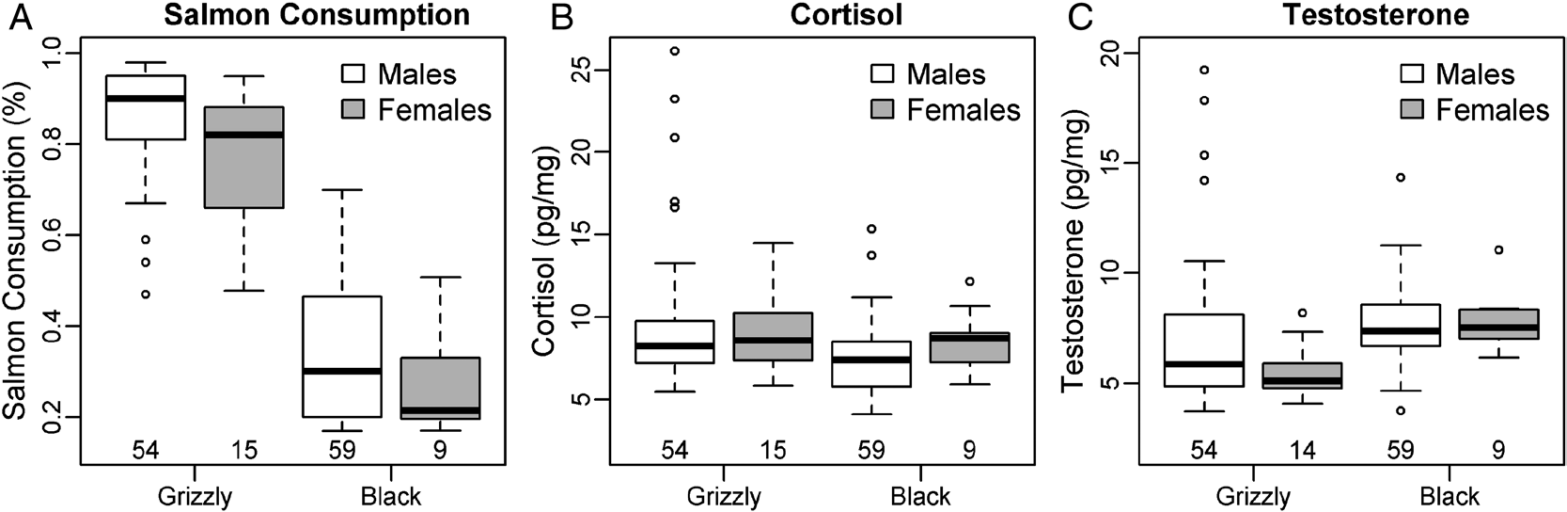
Median salmon consumption (**A**), cortisol (**B**) and testosterone (**C**) based on hair analysis of grizzly and black bears from coastal British Columbia, Canada. Extreme outliers (see Materials and methods; *n* = 5) were excluded; the influence of remaining outliers was reduced by using non-parametric statistics or data transformations, as described in the Materials and methods. Other analyses were performed on grizzly bear data in Bryan *et al.* (2013b).

In our core study area, salmon available in estimated home ranges of both grizzly and black bears generally decreased over the last decade, with an exception in 2009 (Fig. 1A). Salmon consumption by male grizzly bears was higher in 2009 than in 2008 when salmon biomass was lower (*W* = 3, *P*_adj(2)_ = 0.009; Fig. 1B). Although salmon consumption by grizzly bears did not differ between 2009 and 2010 (*W* = 59, *P*_adj(2)_ = 0.97), grizzly bears consumed more variable amounts of salmon in 2010 when biomass was lower (*F*_11,9_ = 4.06, *P* = 0.045). In contrast, salmon consumption by black bears did not vary over time (for 2008–2010, *W* = 176, *P*_adj(2)_ = 0.48; and for 2009–2010, *W* = 210, *P*_adj(2)_ = 0.48; Fig. 1B). The relationships between salmon consumption and availability were non-linear in both species (Fig. 1C and D). Among male grizzly bears, and regardless of year and salmon availability, most bears assimilated >80% of their yearly protein from salmon (Fig. 1C). Among male black bears, salmon consumption was more variable, and all bears assimilated <70% of their yearly protein from salmon. Moreover, salmon consumption generally increased with salmon biomass <200 000 kg and decreased with subsequent increases in biomass (Fig. 1D). Between species, grizzly bears ate more salmon than black bears (for males, *W* = 2393, *P*_adj(4)_ < 0.001; and for females, *W* = 134, *P*_adj(4)_ < 0.001; Fig. 2A). Male grizzly bears ate more salmon than females (*W* = 435, *P*_adj(4)_ = 0.037; Fig. 2A). In black bears, salmon consumption did not differ between sexes (*W* = 326, *P*_adj(4)_ = 0.28; Fig. 2A).

### General trends in cortisol and testosterone

Species and sexes showed notably different hormone levels. Cortisol was higher in grizzly bears compared with black bears, though only in males (*W* = 2115, *P*_adj(4)_ = 0.003; Fig. 2B). In contrast, testosterone was lower in grizzly bears of both sexes (for males, *W* = 1147, *P*_adj(4)_ = 0.021; and for females, *W* = 9, *P*_adj(4)_ = 0.007; Fig. 2C). There were no significant differences in cortisol or testosterone between sexes in grizzly bears (for cortisol, *W* = 373, *P*_adj(4)_ = 1.00; and for testosterone, *W* = 493, *P*_adj(4)_ = 0.22) or black bears (for cortisol, *W* = 167, *P*_adj(4)_ = 0.23; and for testosterone, *W* = 258, *P*_adj(4)_ = 0.90).

### Cortisol

**Figure 3:**
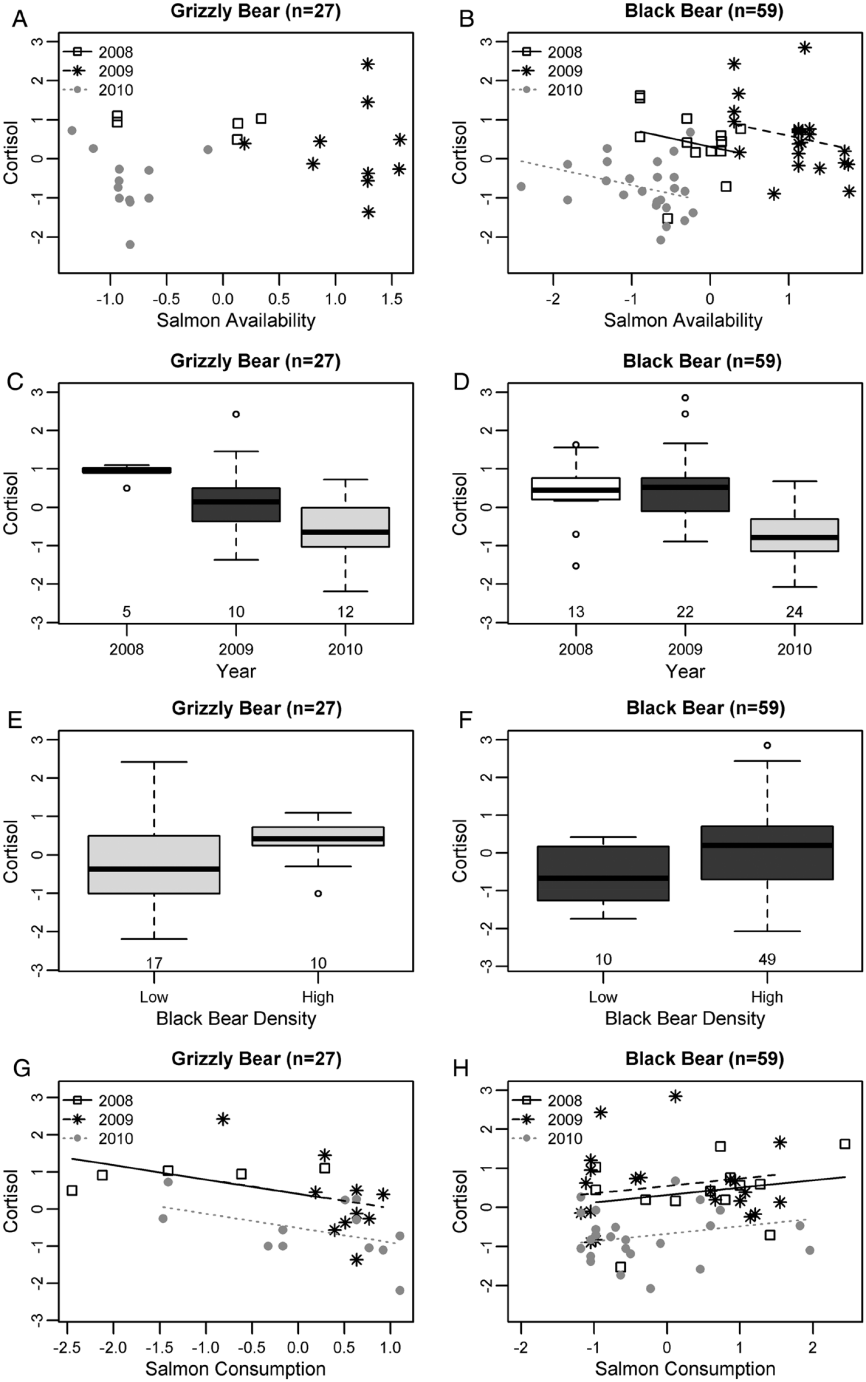
Relationships between cortisol and predictor variables identified by model selection in male grizzly or black bears. (**A**) Salmon availability was not included in the top model set for grizzly bears. (**B**) After controlling for an effect of year, cortisol decreased with increasing salmon availability in black bears. (**C** and **D**) Cortisol showed a similar trend over time in grizzly and black bears, particularly in 2009 and 2010. (**E** and **F**) Both species had higher cortisol in areas with higher black bear densities. (**G** and **H**) Cortisol decreased with increasing salmon consumption in grizzly bears and increased with increasing salmon consumption in black bears. Relationships were predicted from the top model for each species. Other terms in the model were held constant at their mean. Variables were transformed and standardized (see Materials and methods). Other analyses were performed on grizzly bear data in Bryan *et al.* (2013b).

In male grizzly bears, individual variables or a combination of individual and environmental variables best explained hair cortisol levels (Table 3 and Fig. 3). Cortisol decreased with increasing salmon consumption in both the larger data set (*n* = 54) and the subset (*n* = 33) (Table 4 and Fig. 3G). In the larger data set (*n* = 54), cortisol also decreased with increasing watershed productivity, though the relationship was not significant (Table 4). In bears from the smaller core study area, where it was possible to consider year and bear density, both environmental and individual variables were important predictors of cortisol, which was higher in 2009 (corresponding to a low salmon year in the season before hair was grown) and lower in 2010 relative to 2008 (Table 4 and Fig. 3C). Cortisol was higher in areas with higher black bear density and increased with increasing testosterone (Table 4 and Fig. 3E).

**Table 3:** Relative weights (ω) from comparisons of top models in each category (environmental, individual or both) for grizzly and black bears

Response variable	Environment (watershed productivity, salmon availability)	Environment (biomass, year, bear density)	Individual (diet, inter-hormone)	Environment + individual + interactions
Cortisol, grizzly (*n* = 54)	14.7% (4) 0.03	–	48.3% (4) 0.07	37.0% (3) 0.08
Testosterone, grizzly (*n* = 54)	32.9% (4) 0.12	–	4.3% (4) 0	62.8% (11) 0.18
Cortisol, grizzly (*n* = 27)	0.1% (4) 0.03	1.7% (12) 0.28	0.6% (4) 0.19	97.6% (4) 0.59
Testosterone, grizzly (*n* = 27)	5.3% (4) 0.04	3.4% (13) 0.34	6.3% (4) 0.19	85.0% (2) 0.32
Cortisol, black (*n* = 59)	0% (4) 0.10	26.3% (11) 0.44	0% (4) 0.13	73.7% (5) 0.47
Testosterone, black (*n* = 59)	0.1% (4) 0.06	0.1% (11) 0.06	0.9% (4) 0.14	99.0% (7) 0.32

The numbers of models considered for each category are shown in parentheses, and the proportion of variance explained by each model (adjusted *r*^2^) is the third value in each cell. Weights for each top model were calculated by dividing the weight for that model by the sum of weights for the top models from each category.

**Table 4:** Standardized estimates (±SE) for parameters occurring in the top models (ΔAICc ≤ 2) for hair cortisol in male grizzly and black bears

Model set	Model rank	Environment		Environment			Individual		Environment × individual
		Watershed productivity	Salmon availability	Year 2009	Year 2010	Black bear density	Salmon consumption	Testosterone	Availability × consumption
Grizzly (*n* = 54)	1	–	–	–	–	–	−0.30 ± 0.13*	–	–
	2	−0.18 ± 0.13	–	–	–	–	−0.27 ± 0.13*	–	–
Grizzly (*n* = 27)	1	–	–	0.56 ± 0.47	−0.65 ± 0.42	0.84 ± 0.26*	−0.48 ± 0.16*	0.36 ± 0.13*	–
Black (*n* = 59)	1	–	−0.43 ± 0.20*	0.80 ± 0.36*	−1.27 ± 0.29*	–	0.19 ± 0.10	0.27 ± 0.10*	–
	2	–	−0.39 ± 0.20	0.73 ± 0.36*	−1.24 ± 0.29*	0.36 ± 0.26	0.17 ± 0.10	0.24 ± 0.10*	–
	3	–	−0.50 ± 0.21*	0.91 ± 0.38*	−1.24 ± 0.29*	–	0.19 ± 0.10	0.26 ± 0.10*	−0.10 ± 0.11

Grizzly bear data were tested at two spatial scales using a similar set of models: (i) samples collected from the entire coast of British Columbia over 8 years (*n* = 54); and (ii) samples from a subset of grizzly bears (*n* = 27) collected over a smaller area in 3 years, which allowed better comparison with black bear models. Owing to sampling limitations, it was not possible to include year and bear density estimates in the model set for grizzly bears at the larger spatial scale. An intercept-only model was included in all candidate model sets (Tables S4 and S5). Abbreviation: ΔAICc, change in Akaike's information criterion, corrected for small sample size. *Significant at α = 0.05.

As with grizzly bears, black bear cortisol was best explained by both individual and environmental variables; however, the relative importance of environmental variables was higher in black bears (Table 3). Year was the most important predictor of black bear cortisol and showed a similar trend to that in grizzly bears, with cortisol being higher in 2009 and lower in 2010 relative to 2008 (Table 4 and Fig. 3D). Cortisol decreased with salmon availability (Fig. 3B) and increased with testosterone (Table 4). Cortisol was marginally higher in areas with higher black bear density compared with areas of lower black bear density (Table 4 and Fig. 3F). Cortisol increased with salmon consumption in black bears, which is opposite to the trend in grizzly bears (Table 4 and Fig. 3H).

### Testosterone

**Figure 4:**
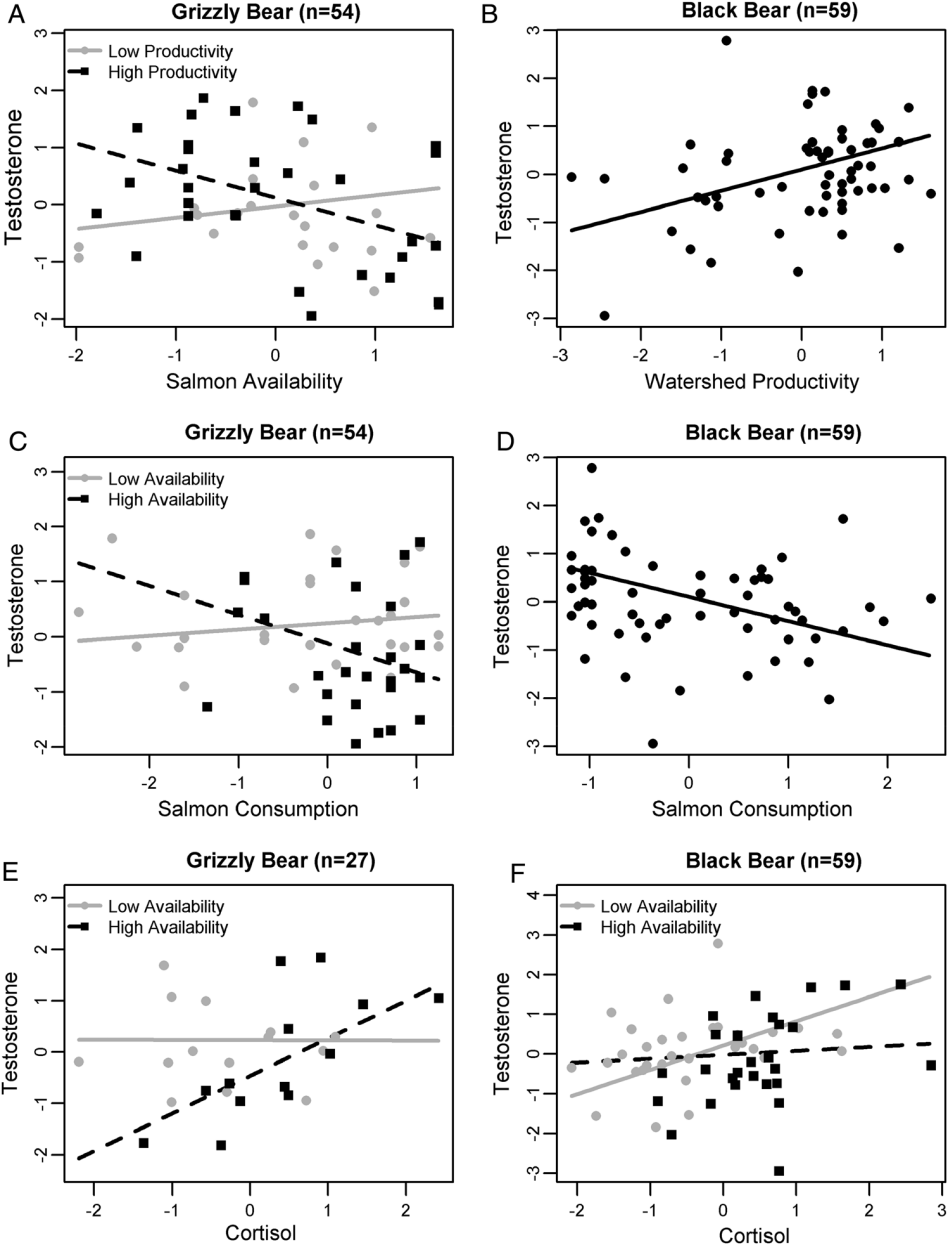
Relationships between testosterone and predictor variables identified by model selection in grizzly or black bears. (**A**) In grizzly bears, testosterone generally decreased with increasing salmon availability in productive and less productive watersheds; however, two bears with low testosterone and low salmon availability appeared to drive a modest positive trend in watersheds with low productivity. (**B**) In black bears, testosterone increased with increasing watershed productivity. (**C**) In grizzly bears, a lack of data on bears with low salmon consumption and high salmon availability caused an interaction between the two variables. (**D**) In black bears, testosterone decreased with increasing salmon consumption. (**E** and **F**) Testosterone and cortisol were positively associated in both grizzly and black bears; however, the strength of the association varied with salmon availability. Relationships were predicted from the top model for each species. Other terms in the model were held constant at their mean. Interacting terms were split into high or low at their mean values. All models were fitted on variables standardized and transformed (see Materials and methods). Other analyses were performed on grizzly bear data in Bryan *et al.* (2013b).

**Figure 5:**
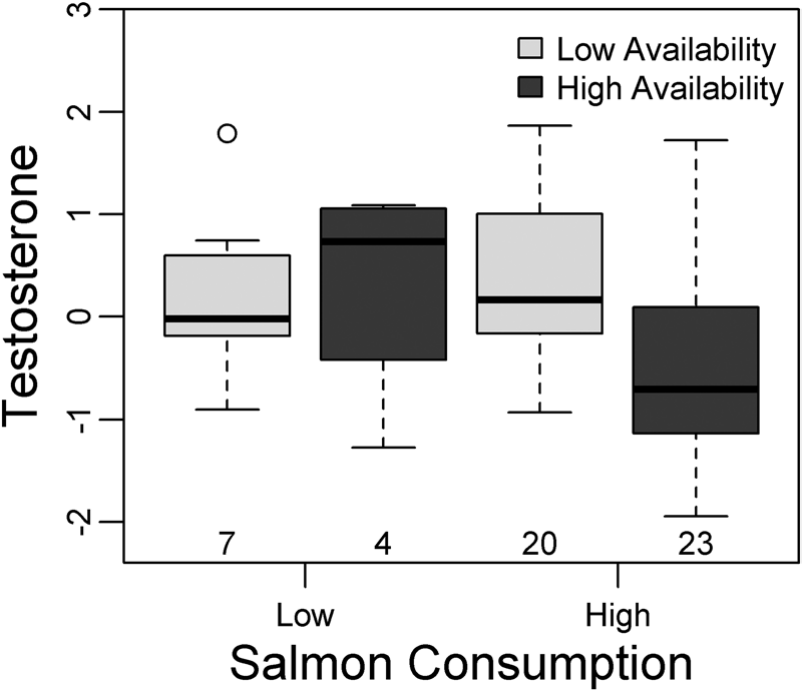
Male grizzly bears that consumed high amounts of salmon had lower testosterone when salmon availability was high compared with when salmon availability was low. Other analyses were performed on these data in Bryan *et al.* (2013b).

In coastal male grizzly bears, testosterone was best predicted by both individual and environmental variables (Table 3). In the larger data set (*n* = 54), testosterone generally decreased with salmon availability, but the relationship differed depending on watershed productivity (Fig. 4A and Table 5). This interaction term was driven by two bears with low testosterone living in watersheds with low productivity (Fig. 4A). Testosterone decreased with increasing salmon consumption, though the relationship differed depending on salmon availability (Table 5). A plot of this interaction term revealed that it was caused by a lack of data on bears with high salmon availability and low consumption, which under-constrained the regression (Fig. 4C). This interaction, however, revealed an interesting trend; bears with high salmon consumption had low testosterone only when salmon availability was high (Fig. 5). In the smaller data set (*n* = 27), there was no interaction between watershed productivity and salmon availability, probably because the two bears described above were not from the core study area (Table 5). Testosterone generally decreased with increasing salmon availability and increased with cortisol. However, the relationship between cortisol and testosterone differed depending on yearly availability of salmon; cortisol and testosterone were more strongly and positively correlated when salmon availability was high (Fig. 4E). Salmon consumption was not included in the top model set at the smaller spatial scale (Table 5).

**Table 5:** Standardized estimates (±SE) for parameters occurring in the top models (ΔAICc ≤ 2) for hair testosterone in male grizzly and black bears

Model set	Model rank	(1) Environment			(2) Individual		(3) Environment × individual	
		Watershed productivity	Salmon availability	Productivity × availability	Salmon consumption	Cortisol	Availability × consumption	Availability × cortisol
Grizzly (*n* = 54)	1	0.09 ± 0.13	−0.22 ± 0.13	−0.39 ± 0.16*	−0.20 ± 0.13	–	−0.38 ± 0.17*	–
	2	0.09 ± 0.13	−0.30 ± 0.13*	−0.36 ± 0.16*	–	–	–	–
Grizzly (*n* = 27)	1	–	−0.39 ± 0.17*	–	–	0.35 ± 0.17	–	0.41 ± 0.16*
Black (*n* = 59)	1	0.40 ± 0.12*	−0.20 ± 0.12	–	−0.49 ± 0.12*	0.39 ± 0.12*	–	−0.23 ± 0.15
	2	0.40 ± 0.12*	–	–	−0.47 ± 0.12*	0.33 ± 0.11*	–	–

Grizzly bear data were tested at two spatial scales using a similar set of models: (i) samples collected from the entire coast of British Columbia over 8 years (*n* = 54); and (ii) samples from a subset of grizzly bears (*n* = 27) collected over a smaller area in 3 years, which allowed better comparison with black bear models. Owing to sampling limitations, it was not possible to include year and bear density estimates in the model set for grizzly bears at the larger spatial scale. An intercept-only model was included in all candidate model sets (Tables S4 and S5). Abbreviation: ΔAICc, change in Akaike's information criterion, corrected for small sample size. *Significant at α = 0.05.

As with grizzly bears, testosterone in male black bears was predicted by both individual and environmental variables (Table 3). In addition, in a similar manner to grizzly bears, testosterone in black bears decreased with salmon availability and increased with cortisol (Table 5). However, a non-significant interaction term (*P* = 0.15) suggested that the relationship between testosterone and cortisol was stronger when salmon availability was low, which was opposite to the trend in grizzly bears (Fig. 4E and F). Moreover, black bear testosterone increased more strongly with watershed productivity compared with grizzly bears (Table 5 and Fig. 4B). The amount of salmon consumed was also included in the top model set; black bears with higher testosterone consumed less salmon (Table 5 and Fig. 4D).

## Discussion

**Figure 6:**
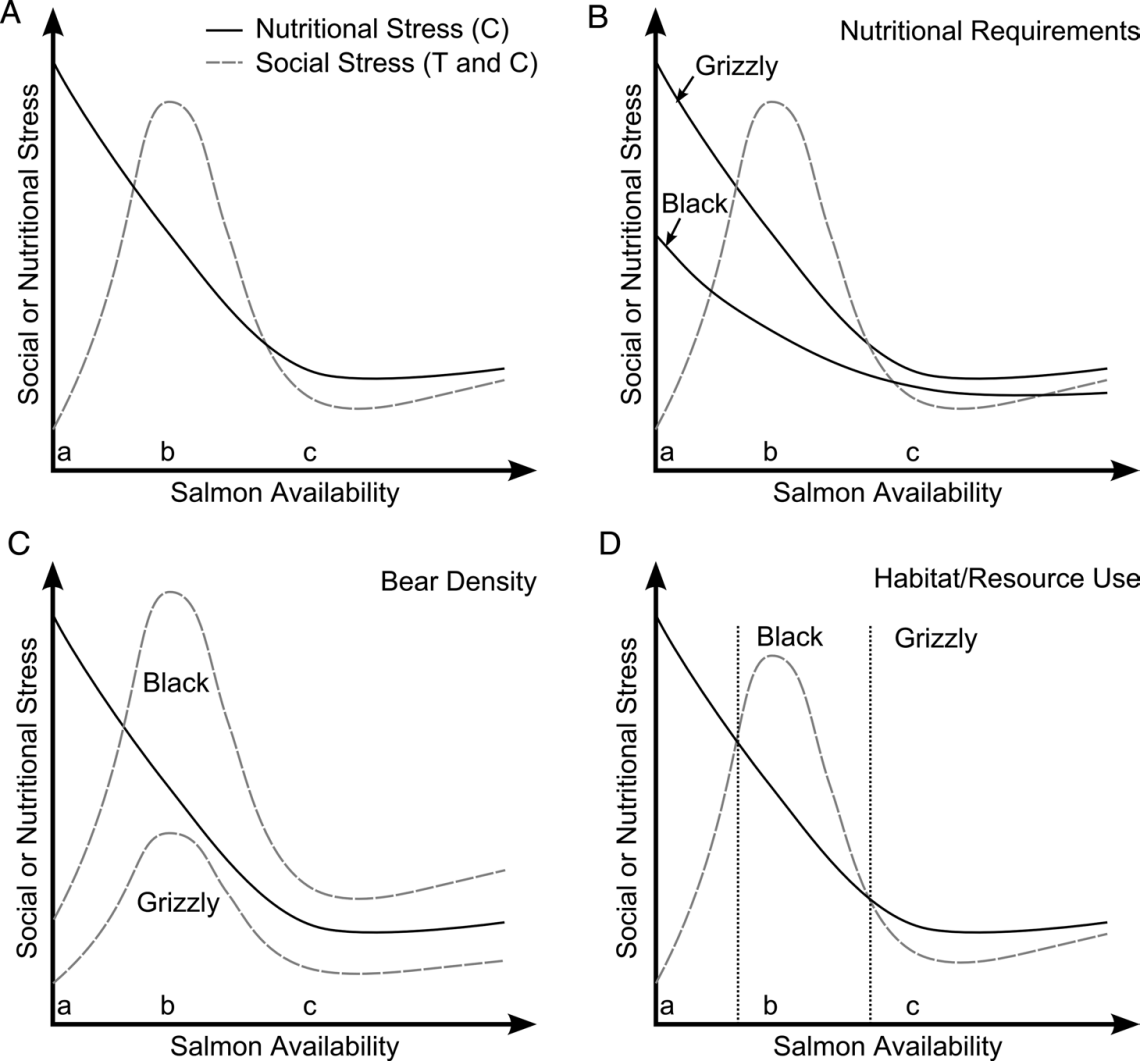
Conceptual model describing how salmon availability might affect social and nutritional stress in coastal grizzly bears and black bears. For simplicity, we assume a population with stable density where salmon availability varies on a yearly basis from low to super-abundant relative to the number of bears. Furthermore, we assume that cortisol represents either social or nutritional stress and that testosterone reflects social stress (i.e. stress due to social conditions). Although many other variables affect testosterone and cortisol, we focus here on the relationship with salmon availability. (**A**) When salmon availability is low (at symbol ‘a’), nutritional stress would be high and social density would be low because bears would use other, more dispersed, resources or move elsewhere to access salmon. When salmon availability is intermediate relative to the number of bears, competition would be high and nutritional stress would be intermediate (symbol ‘b’). Both social and nutritional stress would be low when enough salmon is available that most bears meet their nutritional requirements (symbol ‘c’). We suggest that variations in this model could explain the differences we observed between grizzly and black bears as follows: grizzly and black bears might have different nutritional requirements (**B**), population densities (**C**) or habitat and resource use (**D**). We note that no single model completely explains our data; combinations of these models plus other factors described in the text are probably important.

Based on steroid hormone signals and dietary information incorporated into hair, our data provide novel insight into the mechanisms by which salmon abundance and availability might mediate the social and nutritional contexts of bears. Notably, the non-linear relationships between salmon consumption and salmon biomass in both species provide evidence that the two variables reflect different individual and/or ecological conditions. Correspondingly, testosterone and cortisol were predicted by variables relating to both salmon availability and consumption, with patterns revealing interesting differences between species. Below, we discuss several alternative explanations that are not mutually exclusive for our findings. In addition, we present a conceptual model to aid in visualization of the explanations that we consider most parsimonious for the differences we observed between species (Fig. 6). For simplicity, our model assumes that testosterone will generally reflect social conditions, whereas cortisol will reflect social and nutritional conditions experienced by bears. In brief, our model postulates that cortisol and testosterone vary as a function of salmon availability relative to local bear density. Specifically, social stress should be low and nutritional stress high when salmon availability is low, because bears would leave a watershed or use more dispersed resources. At intermediate numbers of salmon, nutritional stress should be moderate and social stress elevated due to competition for salmon. When salmon are super-abundant, both social and nutritional stress should be low, because most bears are able to meet their nutritional requirements without having to compete for food.

### Cortisol

We hypothesized that cortisol in grizzly bears would increase with decreasing salmon consumption via the following two possible mechanisms: (i) bears that consume less salmon would be nutritionally stressed (an individual variable); and/or (ii) bears experience increased social stress when salmon are less abundant (an environmental variable). Our model selection approach showed that the amount of salmon that grizzly bears consumed—a direct measure of a bear's nutritional context—better predicts cortisol than salmon availability and watershed productivity, which would affect the number of bears present and the type of intra-specific interactions. These findings support our nutritional hypothesis and are consistent with studies attributing elevated cortisol to nutritional stress in other species (Hellgren *et al.*, 1993; Kitaysky *et al.*, 2007; Behie *et al.*, 2010; Ayres *et al.*, 2012). Notably, elevated cortisol could be an adaptive response to food shortages by mobilizing fat (Harlow *et al.*, 1990), by promoting bone resorption (Donahue *et al.*, 2003) or by influencing food-seeking behaviours (Pfeffer *et al.*, 2002; Reneerkens *et al.*, 2002; Pravosudov, 2003) and appetite (Epel *et al.*, 2001).

In contrast to grizzly bears, cortisol in black bears was more strongly predicted by salmon availability than salmon consumption; cortisol was lower when more salmon were available. These findings indicate that cortisol in black bears is influenced more strongly by the social environment mediated by yearly trends in salmon abundance than by the nutritional context alone. Social stress in black bears would occur if lower salmon availability leads to increased real or perceived competition over access to salmon (Dantzer *et al.*, 2013). Although not significant, higher cortisol levels in areas with higher black bear density—where social density would be higher—support this explanation. Interestingly, grizzly bears also had higher cortisol in areas with higher black bear density, which could relate to inter-specific interactions or to habitat differences in areas with higher black bear densities. Among grizzly bears, salmon availability was lower in areas with higher black bear densities, supporting this latter possibility.

Although grizzly and black bears showed different trends in relationship to salmon consumption and availability, both had a similar pattern over time, with higher cortisol after years of low salmon abundance compared with a year of very abundant salmon. Based on our previous findings (Bryan *et al.*, 2013b), we hypothesized that the cortisol differences among years in grizzly bears related to salmon consumption in the previous year. If bears consumed less salmon when less was available in 2007 and 2008, they would have entered hibernation in poorer body condition compared with 2009, which would lead to higher nutritional stress in 2008 and 2009. Changes in salmon consumption over time support this explanation in grizzly bears (Bryan *et al.*, 2013b); however, diet in black bears changed little among years, providing further evidence that different factors influence cortisol in black bears.

Several possibilities could explain the differences we observed between grizzly and black bears. Black bears, which are smaller than grizzly bears, might be better able to meet their energetic requirements from plant-based diets alone and would therefore be less likely to experience nutritional stress (cortisol) due to lower salmon consumption (Welch *et al.*, 1997; Belant *et al.*, 2010; McLellan, 2011). We present this option in Fig. 6B; however, this is unlikely to be the only explanation, because like grizzly bears, black bears can gain energy much more efficiently on a diet of salmon (Welch *et al.*, 1997). Moreover, where the two species are allopatric, black and grizzly bears eat similar amounts of salmon (Jacoby *et al.*, 1999). A more likely explanation for the differences between species is higher social density in black relative to grizzly bears (Fig. 6C). We detected more than twice as many black as grizzly bears in our grid-based study area, a pattern predicted by known differences in densities of both species (Miller *et al.*, 1997).

Social stress in black bears could also be more pronounced because of inter-specific interactions with grizzly bears (Fig. 6D). Grizzly bears have a competitive advantage due to their larger body size (Jacoby *et al.*, 1999; Belant *et al.*, 2006, 2010; Fortin *et al.*, 2007) and occasionally prey on black bears (Mattson *et al.*, 1992; Gunther *et al.*, 2002). To avoid encountering grizzly bears, black bears often use alternative foraging locations or times of day (Machutchon *et al.*, 1998; Belant *et al.*, 2006; Fortin *et al.*, 2007; Schwartz *et al.*, 2010). If these alternative foraging strategies are less profitable or if fishing sites are easier to monopolize, black bears would experience more intense intra-specific social stress related to accessing salmon. Lower salmon consumption among black than grizzly bears supports this possibility. Moreover, black bears might risk exposure to grizzly bears when habitat quality is extremely high (e.g. where salmon is available), which could lead to stress levels induced by fear of predation or interference competition with grizzly bears (Clinchy *et al.*, 2013). Although grizzly bear density was not an important predictor of cortisol in black bears, our density estimates were averaged over space and time and might not reflect densities on salmon spawning streams (see Marerials and methods).

### Testosterone

Patterns in testosterone are consistent with higher social density and/or reproductive activity in black relative to grizzly bears. In grizzly bears, coastal individuals had higher testosterone than non-salmon-eating individuals from the interior, which we attributed to higher social density mediated by salmon availability (Bryan *et al.*, 2013b). Between species, black bears had higher testosterone than grizzly bears. We posit that, similar to density-related testosterone differences among grizzly populations, higher densities or higher reproductive activity in black relative to grizzly bears might explain this trend (Wingfield *et al.*, 1990, 2001; Oliveira, 2004). However, more data are warranted on testosterone levels to determine whether inter-specific patterns could be due to different baseline concentrations, hair structure or reproductive cycles (Palmer *et al.*, 1988; Garshelis and Hellgren, 1994; White *et al.*, 2005). Surprisingly, male bears did not have higher hair testosterone than females in either bear species, probably because of the small sample size for females and our lack of data on age and reproductive status. Moreover, hair reflects testosterone integrated over the breeding and non-breeding seasons in males, when testosterone levels vary (Garshelis and Hellgren, 1994; White *et al.*, 2005), as well as the delayed implantation stage of pregnancy in females, when corpora lutea are capable of producing androgens (Tsubota *et al.*, 1994). Differences in testosterone between sexes might therefore be less distinct in hair than in samples collected at a single time point, such as serum (but see Bryan *et al.*, 2013b).

Within bear species, we used a model selection approach to explore whether testosterone levels varied in relationship to watershed productivity. In particular, we hypothesized that testosterone would increase with watershed productivity, which might reflect higher bear social density or higher reproductive activity in watersheds where more salmon, on average, is available. Consistent with our prediction, testosterone increased with watershed productivity in black bears. In contrast, there was little evidence that testosterone in grizzly bears increased with watershed productivity; possibly because grizzly population density is generally low or grizzly bears consistently occupy productive watersheds where important resources, such as salmon, are sufficiently abundant that bears do not have to compete over them.

We also investigated whether testosterone varied in relationship to salmon availability, which would suggest that elevated testosterone is related to competition for salmon. Based on an established link between fitness and salmon consumption, combined with strong competition observed among bears at spawning salmon streams, we hypothesized that yearly changes in salmon availability would affect the social environment, leading to changes in testosterone. Consistent with our prediction, both bear species showed lower testosterone when more salmon was available, probably reflecting a less competitive social environment when salmon numbers were above average (Fig. 6).

In addition to the environmental variables described above, testosterone was related to individual variables (salmon consumption and cortisol) in both species. We did not detect a positive association between testosterone and salmon consumption in either species, a relationship that would occur if salmon consumption relates to reproductive activity or if bears with high testosterone are more likely to consume salmon. This result, however, must be interpreted with caution for several reasons. First, we did not have data to control for effects of age, social rank, body size or reproductive activity, all of which would be related to salmon consumption and testosterone. Second, our measure of testosterone in hair is integrated over the breeding and non-breeding period in males and might therefore mask trends related directly to reproductive activity. Finally, the majority of salmon consumption would occur after the breeding season in bears; consequently, measuring salmon consumption in longitudinal hair samples collected from the same individuals in different years would provide a better understanding of the timing of any effect of salmon consumption on reproductive activity.

Notably, model selection revealed that testosterone was lower among bears that consumed more salmon in both species. This trend would occur if testosterone levels are lowered by environmental contaminants found in salmon, an effect detected in polar bears (Oskam *et al.*, 2003). Indeed, coastal salmon-consuming grizzly bears have higher levels of persistent organic pollutants than grizzly bears that do not consume salmon (Christensen *et al.*, 2005). However, we found previously that testosterone was higher in coastal salmon-consuming grizzly bears than in a grizzly population that does not consume salmon, suggesting that salmon consumption does not decrease testosterone overall (Bryan *et al.*, 2013b). Moreover, the trend was weaker in grizzly bears than in black bears, even though grizzly bears consumed more salmon. Alternatively, the decreasing trend in testosterone with increasing salmon consumption would occur if bears with low testosterone are consistently more successful at consuming salmon, which might occur if social conditions are stable and dominant bears have low testosterone (Sapolsky, 1993).

Though individual traits would certainly contribute to testosterone levels and should be investigated further, our findings can also be interpreted as further evidence that the social competitive environment is mediated by salmon availability. When salmon is abundant, there would be lower perceived or real competition; consequently, testosterone would be lower and salmon consumption would generally be high (Fig. 6A, symbol ‘c’). In contrast, when competition over salmon is more intense, testosterone would be higher and salmon availability would be lower (Fig. 6A, letter b). An interaction term in the top model for grizzly bears reflected the trend in Fig. 6A (symbol ‘b’) where testosterone was higher among bears with high salmon consumption when salmon availability was low compared with periods of high salmon availability (Fig. 5).

Interestingly, the relationship between testosterone and cortisol varied in magnitude as a function of salmon availability in grizzly bears, as our conceptual model predicts (Fig. 6A, symbols ‘a’ and ‘b’). Specifically, cortisol and testosterone are dissociated at low levels of salmon availability, whereas they are correlated positively when salmon abundance is high. This suggests that nutritional stress overwhelms the signal of social stress when salmon availability is low. In contrast to grizzly bears, there was weak evidence that the relationship between cortisol and testosterone varied in magnitude in relationship to salmon availability in black bears. This provides additional evidence that social density or other stressors are stronger among black bears compared with grizzly bears.

### Conclusion

In conclusion, our findings provide insight into the physiological effects of resource declines on wildlife and could inform future hypothesis testing in this and other systems. In particular, we suggest that parameters in the graphical explanation for our findings would be context dependent, would differ between species, and could be determined empirically with more data. Specifically, future studies could include longitudinal behavioural and ethological observations as well as direct measures of social density, resource availability and resource consumption (e.g. Dantzer *et al.*, 2013). This would enable incorporation of other aspects of salmon availability, such as stream characteristics and the number of fishing sites, as well as information on age, social status, sex ratios and body size of the bears. The inclusion of female bears, which were not well represented at the hair-snag stations, would provide additional needed insight into population-level processes (Bourbonnais *et al.*, 2013).

Our findings also highlight the potential conservation value of fisheries management practices and quotas that ensure adequate salmon for bears and other salmon consumers (Darimont *et al.*, 2010; Levi *et al.*, 2012). Although physiological responses are adaptive mechanisms by which bears cope with environmental heterogeneity (Boonstra, 2013), hormone levels in hair might serve as useful early indicators of the welfare of individuals (Paquet and Darimont, 2010; Malcolm *et al.*, 2013), potential fitness implications (Koren *et al.*, 2011) and population trends (Fefferman and Romero, 2013). Ultimately, the models presented here can serve in monitoring bear populations over time in relationship to salmon declines, especially when strengthened by expansion and corroboration through further research. Moreover, similar approaches may be valuable in elucidating the complex mechanisms by which resource availability and other human disturbances affect physiology, behaviour and population dynamics in a broad array of species and systems.

## Supplementary material

Supplementary material is available at *Conservation Physiology* online.

## Funding

This work was supported by the Raincoast Conservation Foundation; Animal Welfare Institute; Blue Planet Links; Environment Canada Science Horizons; Explorers Club; Habitat Conservation Trust Foundation; Hakai Beach Institute; McLean Foundation; Moore Foundation; Norcross Foundation; National Science and Engineering Council (NSERC) [grant numbers 435683 to C.T.D., RGPIN-106386-2008 to K.E.W.-E. and RGPIN-22876-20 to J.E.G.S.]; Spirit Bear Research Foundation; Tides Canada Wild Salmon Ecosystem Funds; University of Calgary; Willow Grove Foundation; and Wilburforce Foundation. H.M.B. was supported by an NSERC postgraduate Industrial PhD fellowship as well as by the University of Calgary. H.M.B. and C.T.D. recognize support from the Tula Foundation.

## Supplementary Material

Supplementary Data

## References

[1] Anderson DR Burnham KP Thompson WL (2000) Null hypothesis testing: problems, prevalence and an alternative. J Wildl Manag 64: 912–923.

[2] Ayres KL Booth RK Hempelmann JA Koski KL Emmons CK Baird RW Balcomb-Bartok K Hanson MB Ford MJ Wasser SK (2012) Distinguishing the impacts of inadequate prey and vessel traffic on an endangered killer whale (*Orcinus orca*) population. PLoS One 7: e36842. 22701560 10.1371/journal.pone.0036842 PMC3368900

[3] BCGOV (2006) The British Columbia historical fish distribution 50k spatial dataset. Ecosystems Branch, British Columbia Ministry of Environment. http://www.env.gov.bc.ca/esd/distdata/ecosystems/bc50kfiss/hist_fish_dist/

[4] Beckmann JP Berger J (2003) Using black bears to test ideal-free distribution models experimentally. J Mammal 84: 594–606.

[5] Behie AM Pavelka MSM Chapman CA (2010) Sources of variation in fecal cortisol levels in howler monkeys in Belize. Am J Primatol 72: 600–606. 20166191 10.1002/ajp.20813

[6] Belant JL Kielland K Follmann EH Adam LG (2006) Interspecific resource partitioning in sympatric ursids. Ecol Appl 16: 2333–2343. 17205908 10.1890/1051-0761(2006)016[2333:irpisu]2.0.co;2

[7] Belant JL Griffith B Zhang Y Follmann EH Adams LG (2010) Population-level resource selection by sympatric brown and American black bears in Alaska. Polar Biol 33: 31–40.

[8] Ben-David M Titus K Beier LVR (2004) Consumption of salmon by Alaskan brown bears: a trade-off between nutritional requirements and the risk of infanticide? Oecologia 138: 465–474. 14673639 10.1007/s00442-003-1442-x

[9] Boonstra R (2013) Reality as the leading cause of stress: rethinking the impact of chronic stress in nature. Funct Ecol 27: 11–23.

[10] Bourbonnais M Nelson T Cattet M Darimont C Stenhouse G (2013) Spatial analysis of factors influencing long-term stress in the grizzly bear (*Ursus arctos*) population of Alberta, Canada. PLoS One 8: e83768. 24386273 10.1371/journal.pone.0083768 PMC3873976

[11] Bryan HM Adams AG Invik RM Wynne-Edwards KE Smits JE (2013a) Hair is a more repeatable reflection of baseline cortisol than saliva or feces in dogs. J Am Assoc Lab Anim Sci 52: 189–196. 23562104 PMC3624789

[12] Bryan HM Darimont CT Paquet PC Wynne-Edwards KE Smits JE (2013b) Stress and reproductive hormones in grizzly bears reflect nutritional benefits and social consequences of a salmon foraging niche. PLoS One 8: e80537. 24312230 10.1371/journal.pone.0080537 PMC3842319

[13] Christensen JR MacDuffee M Macdonald RW Whiticar M Ross PS (2005) Persistent organic pollutants in British Columbia grizzly bears: consequence of divergent diets. Environ Sci Technol 39: 6952–6960. 16201616 10.1021/es050749f

[14] Clinchy M Sheriff MJ Zanette LY (2013) Predator-induced stress and the ecology of fear. Funct Ecol 27: 56–65.

[15] Cooke SJ Blumstein DT Buchholtz R Caro T Fernández-Juricic E Franklin CE Metcalfe J O'Connor CM Cassady St Clair C Sutherland WJ (2013) Physiology, behavior and conservation. Physiol Biochem Zool 87: 1–14. 24457917 10.1086/671165

[16] Costello CM Creel SR Kalinowski ST Vu NV Quigley HB (2009) Determinants of male reproductive success in American black bears. Behav Ecol Sociobiol 64: 125–134.

[17] Craighead J Sumner J Mitchell J (1995) The Grizzly Bears of Yellowstone. Their Ecology in the Yellowstone Ecosystem, 1959–92. Island Press, Washington, DC.

[18] Creel S Dantzer B Goymann W Rubenstein DR (2013) The ecology of stress: effects of the social environment. Funct Ecol 27: 66–80.

[19] Dantzer B Newman AE Boonstra R Palme R Boutin S Humphries MM McAdam AG (2013) Density triggers maternal hormones that increase adaptive offspring growth in a wild mammal. Science 340: 1215–1217. 23599265 10.1126/science.1235765

[20] Darimont CT Bryan HM Carlson SM Hocking MD MacDuffee M Paquet PC Price MHH Reimchen TE Reynolds JD Wilmers C (2010) Salmon for terrestrial protected areas. Conserv Lett 3: 379–389.

[21] Donahue SW Vaughan MR Demers LM Donahue HJ (2003) Serum markers of bone metabolism show bone loss in hibernating bears. Clin Orthop Relat Res 408: 295–301. 12616074 10.1097/00003086-200303000-00040

[22] Egbert AL Stokes AW (1976) The social behaviour of brown bears on an Alaskan salmon stream. Bears: Their Biol Manage 3: 41–56.

[23] Epel E Lapidus R McEwen B Brownell K (2001) Stress may add bite to appetite in women: a laboratory study of stress-induced cortisol and eating behavior. Psychoneuroendocrinology 26: 37–49. 11070333 10.1016/s0306-4530(00)00035-4

[24] Fefferman NH Romero LM (2013) Can physiological stress alter population persistence? A model with conservation implications. Conserv Physiol 1: doi:10.1093/conphys/cot012. 10.1093/conphys/cot012 PMC4806613 27293596

[25] FOC (2012) NuSeds. Regional Adult Salmon Escapement Database 1950–2010. Fisheries and Oceans Canada, Pacific Biological Station, Nanaimo, BC.

[26] Fortin JK Farley SD Rode KD Robbins CT (2007) Dietary and spatial overlap between sympatric ursids relative to salmon use. Ursus 18: 19–29.

[27] Garshelis DL Hellgren EC (1994) Variation in reproductive biology of male black bears. J Mammal 75: 175–188.

[28] Gende SM Quinn TP (2004) The relative importance of prey density and social dominance in determining energy intake by bears feeding on Pacific salmon. Can J Zool 82: 75–85.

[29] Gill I Helfield J (2012) Alternative foraging strategies among bears fishing for salmon: a test of the dominance hypothesis. Can J Zool 90: 766–775.

[30] Groot C Margolis L (1991) Pacific Salmon Life Histories. University of British Columbia Press, Vancouver, BC.

[31] Gunther KA Biel MJ Anderson N Waits L (2002) Probable grizzly bear predation on an American black bear in Yellowstone National Park. Ursus 13: 372–374.

[32] Harlow HJ Beck TDI Walters LM Greenhouse SS (1990) Seasonal serum glucose, progesterone, and cortisol levels of black bears (*Ursus americanus*). Can J Zool 68: 183–187.

[33] Hellgren EC Rogers L Seal U (1993) Serum chemistry and hematology of black bears: physiological indices of habitat quality or seasonal patterns? J Mammal 74: 304–315.

[34] Herrero S (1983) Social behaviour of black bears at a garbage dump in Jasper National Park. Bears: Their Biol Manage 5: 54–70.

[35] Hilderbrand GV Farley S Robbins C Hanley T Titus K Servheen C (1996) Use of stable isotopes to determine diets of living and extinct bears. Can J Zool 74: 2080–2088.

[36] Hilderbrand GV Schwartz CC Robbins CT Jacoby ME Hanley TA Arthur SM Servheen C (1999) The importance of meat, particularly salmon, to body size, population productivity, and conservation of North American brown bears. Can J Zool 77: 132–138.

[37] Hilderbrand GV Schwartz CC Robbins CT Hanley TA (2000) Effect of hibernation and reproductive status on body mass and condition of coastal brown bears. J Wildl Manag 64: 178–183.

[38] Hofer H East ML (2012) Stress and immunosuppression as factors in the decline and extinction of wildlife populations: concepts, evidence and challenges. In Aguirre AA Ostfeld RS Daszak P, eds, New Directions in Conservation Medicine: Applied Cases of Ecological Health. Oxford University Press, New York, pp 109–133.

[39] Jacoby ME Hilderbrand GV Servheen C Schwartz CC Arthur SM Hanley TA Robbins CT Michener R (1999) Trophic relations of brown and black bears in several western North American ecosystems. J Wildl Manag 63: 921–929.

[40] Janson CH van Schaik CP (1988) Recognizing the many faces of primate food competition: methods. Behaviour 105: 165–186.

[41] Jones ES Heard DC Gillingham MP Bowman J (2006) Temporal variation in stable carbon and nitrogen isotopes of grizzly bear guardhair and underfur. Wildl Soc Bull 34: 1320–1325.

[42] Keckeis K Lepschy M Schöpper H Moser L Troxler J Palme R (2012) Hair cortisol: a parameter of chronic stress? Insights from a radiometabolism study in guinea pigs. J Comp Physiol B 182: 985–996. 22592890 10.1007/s00360-012-0674-7

[43] Kempenaers B Peters A Foerster K (2008) Sources of individual variation in plasma testosterone levels. Philos Trans R Soc Lond B Biol Sci 363: 1711–1723. 18048297 10.1098/rstb.2007.0001 PMC2367619

[44] Kendall KC Stetz JB Roon DA Waits LP Boulanger JB Paetkau D (2008) Grizzly bear density in Glacier National Park, Montana. J Wildl Manag 72: 1693–1705.

[45] Kitaysky A Piatt J Wingfield J (2007) Stress hormones link food availability and population processes in seabirds. Mar Ecol Prog Ser 352: 245.

[46] Koenig A Borries C (2006) The predictive power of socioecological models: a reconsideration of resource characteristics, agonism and dominance hierarchies. In Hohmann G Robbins MM Boesch C, eds, Feeding Ecology in Apes and Other Primates. Ecological, Physical and Behavioral Aspects. Cambridge University Press, New York, pp 263–284.

[47] Koren L Mokady O Karaskov T Klein J Koren G Geffen E (2002) A novel method using hair for determining hormonal levels in wildlife. Anim Behav 63: 403–406.

[48] Koren L Nakagawa S Burke T Soma KK Wynne-Edwards KE Geffen E (2011) Non-breeding feather concentrations of testosterone, corticosterone and cortisol are associated with subsequent survival in wild house sparrows. Proc Biol Sci 279: 1560–1566. 22090380 10.1098/rspb.2011.2062 PMC3282351

[49] Kovach AI Powell RA (2003) Effects of body size on male mating tactics and paternity in black bears, Ursus americanus. Can J Zool 81: 1257–1268.

[50] Levi T Darimont CT MacDuffee M Mangel M Paquet P Wilmers CC (2012) Using grizzly bears to assess harvest-ecosystem tradeoffs in salmon fisheries. PLoS Biol 10: e1001303. 22505845 10.1371/journal.pbio.1001303 PMC3323506

[51] Macbeth BJ Cattet MRL Stenhouse GB Gibeau ML Janz DM (2010) Hair cortisol concentration as a noninvasive measure of long-term stress in free-ranging grizzly bears (*Ursus arctos*): considerations with implications for other wildlife. Can J Zool 88: 935–949.

[52] Macbeth BJ Cattet MRL Obbard ME Middel K Janz DM (2012) Evaluation of hair cortisol concentration as a biomarker of long-term stress in free-ranging polar bears. Wildl Soc Bull 36: 747–758.

[53] McCune B Grace GB Urban DL (2002) Analysis of Ecological Communities. MjM Software Design, Gleneden Beach, OR.

[54] Machutchon AG Himmer S Bryden CA (1993). Khutzeymateen Valley Grizzly Bear Study Final Report. BC Ministry of Environment, Lands and Parks, Wildlife Report No. R-25 and BC Ministry of Forests, Wildlife Habitat Research Report No. WHR-31. Victoria, Canada 105 pp. http://www.for.gov.bc.ca/hfd/pubs/docs/mr/Whr31.htm

[55] Machutchon AG Himmer S Davis H Gallagher M (1998) Temporal and spatial activity patterns among coastal bear populations. Ursus 10: 539–546.

[56] McLellan B (2011) Implications of a high-energy and low-protein diet on the body composition, fitness, and competitive abilities of black (*Ursus americanus*) and grizzly (*Ursus arctos*) bears. Can J Zool 89: 546–558.

[57] McMillin JM Seal U Rogers L Erickson A (1976) Annual testosterone rhythm in the black bear (*Ursus americanus*). Biol Reprod 15: 163–167. 963146 10.1095/biolreprod15.2.163

[58] Malcolm K McShea W Van Deelen T Bacon H Liu F Putman S Zhu X Brown J (2013) Analyses of fecal and hair glucocorticoids to evaluate short- and long-term stress and recovery of Asiatic black bears (*Ursus thibetanus*) removed from bile farms in China. Gen Comp Endocrinol 185: 97–106. 23416358 10.1016/j.ygcen.2013.01.014

[59] Mattson DJ Reinhart DP (1995) Influences of cutthroat trout (*Oncorhynchus clarki*) on behaviour and reproduction of Yellowstone grizzly bears (*Ursus arctos*), 1975–1989. Can J Zool 73: 2072–2079.

[60] Mattson DJ Knight RR Blanchard BM (1992) Cannibalism and predation on black bears by grizzly bears in the Yellowstone Ecosystem, 1975–1990. J Mammal 73: 422–425.

[61] Meyer JS Novak MA (2012) Hair cortisol: a novel biomarker of hypothalamic-pituitary-adrenocortical activity. Endocrinology 153: 4120–4127. 22778226 10.1210/en.2012-1226 PMC3423616

[62] Miller SD White GC Sellers RA Reynolds HV Schoen JW Titus K Barnes VG Jr Smith RB Nelson RR Ballard WB (1997) Brown and black bear density estimation in Alaska using radiotelemetry and replicated mark-resight techniques. Wildl Monogr 133: 3–55.

[63] Moore JW Semmens BX (2008) Incorporating uncertainty and prior information into stable isotope mixing models. Ecol Lett 11: 470–480. 18294213 10.1111/j.1461-0248.2008.01163.x

[64] Noakes DJ Beamish RJ Kent ML (2000) On the decline of Pacific salmon and speculative links to salmon farming in British Columbia. Aquaculture 183: 363–386.

[65] Oliveira RF (2004) Social modulation of androgens in vertebrates: mechanisms and function. Adv Study Behav 34: 165–239.

[66] Oskam IC Ropstad E Dahl E Lie E Derocher AE Wiig Ø Larsen S Wiger R Skaare JU (2003) Organochlorines affect the major androgenic hormone, testosterone, in male polar bears (*Ursus maritimus*) at Svalbard. J Toxicol Environ Health A 66: 2119–2139. 14710596 10.1080/15287390390211342

[67] Palmer S Nelson R Ramsay M Stirling I Bahr J (1988) Annual changes in serum sex steroids in male and female black (*Ursus americanus*) and polar (*Ursus maritimus*) bears. Biol Reprod 38: 1044–1050. 3408772 10.1095/biolreprod38.5.1044

[68] Paquet PC Darimont CT (2010) Wildlife conservation and animal welfare: two sides of the same coin. Anim Welf 19: 177–190.

[69] Peterman RM Dorner B Rosenfeld JS (2012) A widespread decrease in productivity of sockeye salmon (*Oncorhynchus nerka*) populations in western North America. Can J Fish Aquat Sci 69: 1255–1260.

[70] Pfeffer K Fritz J Kotrschal K (2002) Hormonal correlates of being an innovative greylag goose, *Anser anser*. Anim Behav 63: 687–695.

[71] Pragst F Balikova MA (2006) State of the art in hair analysis for detection of drug and alcohol abuse. Clin Chim Acta 370: 17–49. 16624267 10.1016/j.cca.2006.02.019

[72] Pravosudov VV (2003) Long-term moderate elevation of corticosterone facilitates avian food-caching behaviour and enhances spatial memory. Proc Biol Sci 270: 2599–2604. 14728783 10.1098/rspb.2003.2551 PMC1691552

[73] Price MHH Darimont CT Temple NF MacDuffee SM (2008) Ghost runs: management and status assessment of Pacific salmon (*Oncorhynchus* spp.) returning to British Columbia's central and north coasts. Can J Fish Aquat Sci 65: 2712–2718.

[74] Proctor M McLellan B Boulanger J Apps C Stenhouse G Paetkau D Mowat G (2010) Ecological investigations of grizzly bears in Canada using DNA from hair, 1995–2005: a review of methods and progress. Ursus 21: 169–188.

[75] R Development Core Team (2011) R: A Language and Environment for Statistical Computing. R Foundation for Statistical Computing, Vienna, Austria ISBN 3-900051-07-0. http://www.R-project.org

[76] Reeder DM Kramer KM (2005) Stress in free-ranging mammals: integrating physiology, ecology, and natural history. J Mammal 86: 225–235.

[77] Reimchen T (2000) Some ecological and evolutionary aspects of bear–salmon interactions in coastal British Columbia. Can J Zool 78: 448–457.

[78] Reneerkens J Piersma T Ramenofsky M (2002) An experimental test of the relationship between temporal variability of feeding opportunities and baseline levels of corticosterone in a shorebird. J Exp Zool 293: 81–88. 12115922 10.1002/jez.10113

[79] Rogers LL (1987) Effects of food supply and kinship on social behavior, movements, and population growth of black bears in northeastern Minnesota. Wildl Monogr 97: 3–72.

[80] Romero LM (2004) Physiological stress in ecology: lessons from biomedical research. Trends Ecol Evol 19: 249–255. 16701264 10.1016/j.tree.2004.03.008

[81] Ruggerone GT Peterman RM Dorner B Myers KW (2010) Magnitude and trends in abundance of hatchery and wild pink salmon, chum salmon, and sockeye salmon in the North Pacific Ocean. Mar Coast Fish 2: 306–328.

[82] Sapolsky RM (1993) The physiology of dominance in stable versus unstable social hierarchies. In Mason WA Mendoza SP, eds, Primate Social Conflict. State University of New York Press, Albany, NY, pp 171–204.

[83] Sapolsky RM Romero LM Munck AU (2000) How do glucocorticoids influence stress responses? Integrating permissive, suppressive, stimulatory, and preparative actions. Endocr Rev 21: 55–89. 10696570 10.1210/edrv.21.1.0389

[84] Schwartz CC Miller SD Haroldson MA (2003) Grizzly Bear. In Feldhamer GA Thompson BC Chapman JA, eds, Wild Mammals of North America: Biology, Management and Conservation, Ed 2 John Hopkins University Press, Baltimore, MD, pp 556–586.

[85] Schwartz CC Cain SL Podruzny S Cherry S Frattaroli L (2010) Contrasting activity patterns of sympatric and allopatric black and grizzly bears. J Wildl Manag 74: 1628–1638.

[86] Seebacher F Franklin CE (2012) Determining environmental causes of biological effects: the need for a mechanistic physiological dimension in conservation biology. Philos Trans R Soc Lond B Biol Sci 367: 1607–1614. 22566670 10.1098/rstb.2012.0036 PMC3350663

[87] Semmens BX Moore JW (2008) MixSIR: A Bayesian Stable Isotope Mixing Model. Version 1.0. http://www.ecologybox.org

[88] Stetz J Hunt K Kendall KC Wasser SK (2013) Effects of exposure, diet, and thermoregulation on fecal glucocorticoid measures in wild bears. PLoS One 8: e55967. 23457488 10.1371/journal.pone.0055967 PMC3573068

[89] Tsubota T Nitta H Osawa Y Mason JI Kita I Tiba T Bahr JM (1994) Immunolocalization of steroidogenic enzymes P450scc, 3βHSD, P450c17 and P450arom in the corpus luteum of the Hokkaido brown bear (*Ursus arctos yesoensis*) in relation to delayed implantation. J Reprod Fertil 101: 557–561. 7966008 10.1530/jrf.0.1010557

[90] van Schaik CP (1989) The ecology of social relationships amongst female primates. In Standen V Foley RA, eds, Comparative Socioecology: the Behavioural Ecology of Humans and Other Mammals. Blackwell Scientific Publications, Oxford, pp 195–218.

[91] Wasser SK Davenport B Ramage ER Hunt KE Parker M Clarke C Stenhouse G (2004) Scat detection dogs in wildlife research and management: application to grizzly and black bears in the Yellowhead Ecosystem, Alberta, Canada. Can J Zool 82: 475–492.

[92] Welch CA Keay J Kendall KC Robbins CT (1997) Constraints on frugivory by bears. Ecology 78: 1105–1119.

[93] White DJ Berardinelli JG Aune KE (2005) Seasonal differences in spermatogenesis, testicular mass and serum testosterone concentrations in the grizzly bear. Ursus 16: 198–207.

[94] Wikelski M Cooke SJ (2006) Conservation physiology. Trends Ecol Evol 21: 38–46. 16701468 10.1016/j.tree.2005.10.018

[95] Wingfield JC Hegner RE Dufty AM Jr Ball GF (1990) The “Challenge Hypothesis”: theoretical implications for patterns of testosterone secretion, mating systems, and breeding strategies. Am Nat 136: 829–846.

[96] Wingfield JC Lynn S Soma KK (2001) Avoiding the ‘costs’ of testosterone: ecological bases of hormone-behavior interactions. Brain Behav Evol 57: 239–251. 11641561 10.1159/000047243

[97] Zedrosser A Bellemain E Taberlet P Swenson JE (2007) Genetic estimates of annual reproductive success in male brown bears: the effects of body size, age, internal relatedness and population density. J Anim Ecol 76: 368–375. 17302844 10.1111/j.1365-2656.2006.01203.x

[98] Zuur AF Ieno EN Walker NJ Saveliev AA Smith GM (2009) Mixed Effects Models and Extensions in Ecology with R. Springer Science + Business Media, New York.

