# Supplementary material for: Stress and reproductive hormones reflect inter-specific social and nutritional conditions mediated by resource availability in a bear–salmon system
Source: Conserv Physiol. 2014 May 2;2(1):cou010. doi: 10.1093/conphys/cou010 (PMC4806744; doi:10.1093/conphys/cou010)
Supplement: Supplementary Data [file supp_2_1_cou010__index.html]

Stress and reproductive hormones reflect inter-specific social and nutritional conditions mediated by resource availability in a bear–salmon system — Supplementary Data 

# Stress and reproductive hormones reflect inter-specific social and nutritional conditions mediated by resource availability in a bear–salmon system

## Supplementary Data

Supplementary Data

**Files in this Data Supplement:**

- Supplementary Data - Doc file
