# Supplementary material for: Stress and reproductive hormones reflect inter-specific social and nutritional conditions mediated by resource availability in a bear–salmon system
Source: Conserv Physiol. 2014 May 2;2(1):cou010. doi: 10.1093/conphys/cou010 (PMC4806744; doi:10.1093/conphys/cou010)

**Supplementary Information for**

**Stress and reproductive hormones reflect inter-specific social and nutritional conditions mediated by resource availability in a bear-salmon system**

Heather M. Bryan^*^, Chris T. Darimont, Paul C. Paquet, Katherine E. Wynne-Edwards,

Judit E. G. Smits

^*^Corresponding author: hmbryan@uvic.ca

**This file includes:**

Supplementary Methods

Measurement of hormone levels in hair

Salmon consumption estimates

Bear density estimates

Salmon data imputations

Salmon biomass calculations

Model sets and model validations for variables explaining cortisol and testosterone in hair from male grizzly and black bears

Figures S1 to S6

Tables S1 to S9

References

*Measurement of hormone levels in hair*

We analyzed cortiosl and testosterone in grizzly and black bear hair collected from the field and in grizzly bear hair from government archives (Table 2). Hair samples from our field collections were genetically linked to individual bears, including their sex and species, using seven microsatellite markers at a commercial laboratory (Wildlife Genetics International). During field sampling between 2009 and 2011, we detected 50 grizzly bears (unique individuals or individuals captured in different years) and 116 black bears. Of these detections, there was adequate hair to perform hormonal assays on 66% of grizzly samples (n=33) and 59% of black bear samples (n=68).

All hair samples were collected over a short period (approximately 4 weeks) in spring during the annual moult. Therefore, the hair we collected represented diet and hormone levels incorporated over an approximately six-month period in the previous year when the hair was growing (Christens*en et a*l., 2005; Darimont and Reimchen, 2002). Single hair samples consisted of <1 to >100 mg of hair. Therefore, we pooled samples obtained from the same individual in the same year to obtain enough material for hormonal (min. 30 mg) and stable isotope analyses (min. 1 mg). Many of the pooled samples came from a single “capture” event (i.e., hair snag); however we also pooled samples collected from the same individual at different locations or dates. Samples were not pooled across years; five grizzly and nine black bear males were sampled in more than one year (Table 2). All hair samples came from unknown body regions. Though hormone levels do vary over different body regions (Macbe*th et a*l., 2010), we did not expect a directional bias. Moreover, we found that measurements of cortisol were correlated and consistent in dog hair collected from different body regions over a three-month period (Bry*an et a*l., 2013a).

We quantified cortisol and testosterone in hair using previously validated enzyme immunoassays (Bry*an, et a*l., 2013a; Bry*an et a*l., 2013b). First, we performed a wash procedure to remove substances such as blood, saliva, or feces, which contain high levels of steroids and could potentially contaminate hair samples. Accordingly, we placed approximately 50 mg of hair in a petri dish and washed each sample twice with 40 mL of tap water and twice with 40 mL of HPLC-grade isopropyl alcohol (Fisher Scientific) for 3 min per wash while rotating at 130 rpm. We thoroughly dried the samples after each set of washes, powdered the hair (25 Hz, Retsch Mixer Mill 200), and weighed 30 mg of powder into a glass scintillation vial. To extract steroids, we added HPLC grade methanol (Omnisolv, VWR) at a concentration of 100 μL per mg of hair powder, sonicated the samples for 30 minutes, and incubated them for 18 hours at 60ºC on a plate rotating at 160 rpm. Next, we centrifuged the samples (30 minutes at 4000 rpm), aliquotted the supernatant into separate tubes for cortisol (2000 μL) and testosterone (100 μL) assays, and evaporated the methanol in a sample concentrator (Techne) using a gentle stream of nitrogen. We reconstituted the samples (5% methanol, 95% assay diluent) and measured immunoreactivity to cortisol and testosterone using salivary immunoassay kits (Salimetrics, Philadelphia, Pennsylvania). Hair extracts were randomly assigned to immunoassay plates (n=15) based on species and sex. Where possible, samples from the same individual in different years were assayed on the same plate. Cross-reactivity of the assays to non-target compounds as well as recovery, repeatability, linearity and inter- and intra-assay variability assessed using bear hair have been previously reported (Bry*an, et a*l., 2013a; Bry*an, et a*l., 2013b). Notably, intra- and inter-assay coefficients of variability for quality controls were all <15% and most were <10% (Bry*an, et a*l., 2013b). As an additional measure of assay performance specifically for black bear hair, we tested whether black bear hair extracts showed a linear dilution compared with assay standards. Analyses of covariance for each assay were non-significant (cortisol p=0.22; testosterone p=0.94), reflecting linearity of the diluted hair extracts.

*Salmon consumption estimates*

In preparation for stable isotope analysis, hair samples were washed with distilled water followed by a 2:1 chloroform:methanol solution. Dried hair samples were then powdered and weighed, as previously described (Darimo*nt et a*l., 2008; Darimo*nt et a*l., 2007; Darimont and Reimchen, 2002) . Next, the ratios of carbon (C^13^/C^12^) and nitrogen (N^15^/N^14^) stable isotopes were quantified using gas chromatography coupled with mass spectrometry at the University of Saskatchewan Stable Isotope Facility. We used Bayesian isotope mixing models to estimate the proportion of a bear’s yearly diet assimilated from salmon (i.e., its ‘salmon consumption’) using Bayesian isotope mixing models (Moore and Semmens, 2008; Semmens and Moore, 2008). The models incorporate isotopic signatures of the samples, isotopic signatures of dietary sources (i.e., salmon and plants), fractionation of isotopes in hair, as well as uncertainties associated with these estimates. For each individual, the model generates a posterior probability distribution for the estimated contributions of each dietary source to the diet; we selected the median estimate as the proportion of each source (plants or salmon) in an individual bear’s diet. Based on a comprehensive analysis of grizzly bear diets from across North America, we assumed that coastal bears consume only plants or salmon (Mowat and Heard, 2006). Accordingly, we used previously published isotopic signatures of salmon and plant dietary baselines, and fractionation rates as inputs in our models (Mowat and Heard, 2006).

*Bear density estimates*

Within the grid-based coastal study area in which 27 of 54 grizzlies and all 59 black bears were sampled, we classified grizzly and black bear densities as high or low. This classification was based on the midpoint of the range in our proxies for density, which were number of unique individuals detected per hair snagging station in 2010 and 2011 averaged over all hair snagging stations within each of 9 conservation landscape units of 342-900 km^2^ (Bry*an, et a*l., 2013b). Thus, our density estimates provided relative estimates of bear density generalized over space and time. Each of 9 land units had a median of 8 hair snags (range: 6-10). Within these land units, the average number of grizzly bears detected per snag station ranged from 0 to 1.1 (median 0.1) and the average number of black bears per hair snag ranged from 0.2 to 0.9 (median 0.7).

*Salmon data imputations*

We estimated the salmon biomass available to grizzly and black bears using salmon enumeration data from Fisheries and Oceans Canada 2010 (FOC, 2012). This database, which is geo-referenced to salmon enumeration points, provides yearly abundance estimates for the 5 Pacific salmon species spawning in streams throughout British Columbia (BC). Though the records are current to 2010, only 8% of runs are monitored consistently (Pri*ce et a*l., 2008). Therefore, we developed an imputation method for missing values (Ruggero*ne et a*l., 2010). We first excluded all species-watershed combinations (69% of 2680) that had been monitored less than five times in the last 15 years, which are typically small runs (Pri*ce, et a*l., 2008). We treated even and odd-year runs of pink salmon as separate species due to their distinct two-year lifecycle; our criterion for excluding even or odd pink salmon runs was less than three observations in the last 15 years. After applying these exclusion criteria and considering only fisheries management areas overlapping our study area, approximately 30% of 12 615 possible watershed-species-year abundance estimates were missing.

We estimated a missing fish count in a given watershed, year, and fisheries management area by 1) computing the long-term relative contributions of all watersheds to the total count for that species in the corresponding fisheries management area, 2) computing an estimate of the total count for that species in that fisheries management area in that year as the average of the ratios of each of the available watershed counts in that year to the long-term relative contributions of those watersheds to counts for that species in that fisheries management area, and 3) imputing the missing fish count from the estimated total count for that fisheries management area by multiplying by the long-term relative contribution of the watershed to that species in that fisheries management area. An example calculation for a hypothetical missing data point (x_1_) is presented in Table S1 and Equation S1. Imputations and validations were performed in R (R Development Core Team, 2011).

To validate this approach, we randomly removed 5% of the existing data, performed imputations and then compared the imputed to existing values using linear regression and Bland-Altman plots. Initial results showed that salmon abundance estimates were overestimated at lower abundance values (data not shown). This bias is expected because the Fisheries and Oceans Canada is less likely to regularly monitor smaller watersheds, particularly in years of low salmon abundance (Pri*ce, et a*l., 2008). Accordingly, we applied a variable correction factor to species-watershed combinations based on the number of missing data points (Fig. S2). The correction factor was fully determined by two parameters: 1) the number of missing values after which threshold a correction factor was applied to account for overestimation bias (N_c_) and 2) the magnitude of the correction (M_c_). We tested combinations of these two parameters and found that values of N_c_=15/4 and M_c_=0.4 adequately mitigated overestimation of imputed values in watersheds with low salmon abundance. Using this function, we applied a correction factor if there were four or more missing values and varied the magnitude to a maximum of 0.4 when there were 10 missing values. For even and odd-year pink salmon, we used an adjusted N_c_=15/8.

After applying the correction factor, Bland-Altman plots showed that the mean difference between existing and imputed data points fell close to zero, reflecting no clear bias in the imputed values (Fig. S1.A). Moreover, most imputed values (94.1%) fell within two standard deviations of the mean difference. Visual inspection of the outliers revealed no consistent trend in species, fisheries area or watershed. Linear regression showed that the imputations provide a good reflection of actual salmon abundance (Fig. S1.B). The average coefficient of variation based on five randomly dropped subsets of existing data was 12.2%; however, we caution that imputation errors would have been compounded if data were missing for multiple species from the same watershed in a given year.

We calculated salmon biomass in a watershed by multiplying fish counts for each species by their average masses (in kg) and summing across all species present. For each species, we used average mass reported in Groot and Margolis (1991), and assumed a 1:1 sex ratio. Following the biomass calculations, we imported the data into ArcGIS 9.3 (ESRI, Redlands, California, USA). There, we joined salmon enumeration points to their corresponding spawning salmon stream data (BCGOV, 2006). We then divided salmon biomass by stream length to be used in placing buffers around bear detection points. We assumed that salmon density would be even along the length of the spawning portions of each stream.

**Table S1.** Example imputation calculation for a hypothetical data point (x_1_) for a given salmon species, watershed, and year.

| **Watershed** | **Year1** | **Year2** | **Year3** | **Year4** | **Watershed Average** | **Average contribution (%)** |
| --- | --- | --- | --- | --- | --- | --- |
| 1 | x_1_ | 10 | 30 | 5 | 15 | 0.4 |
| 2 | 50 | 100 | x_2_ | x_3_ | 75 | 2.1 |
| 3 | 1000 | 1500 | 700 | 2000 | 1300 | 37.2 |
| 4 | 100 | 150 | 500 | x_4_ | 283 | 8.1 |
| 5 | 2000 | 1800 | 2500 | 1000 | 1825 | 52.2 |
| **Total** |  |  |  |  | **3498** | **100%** |

Eq (S1) $x_{1}=0.4\times\frac{\frac{50}{2.1}+\frac{1000}{37.2}+\frac{100}{8.1}+\frac{2000}{52.2}}{4}=10.1$

Figure S1 Validations of the imputation method for missing salmon data after applying a correction factor to account for overestimation.

(A) Bland-Altman plot showing existing data against the difference between existing and imputed data points from a randomly excluded sample of 5% from all species, years, and watersheds. The solid line shows the mean difference and the dashed lines represent two standard deviations from the mean. Values falling above the mean are underestimated and those falling below are overestimated. Points outside two standard deviations are considered outliers. (B) There was a strong linear relationship between existing and imputed data. Salmon abundance data were natural log-transformed.


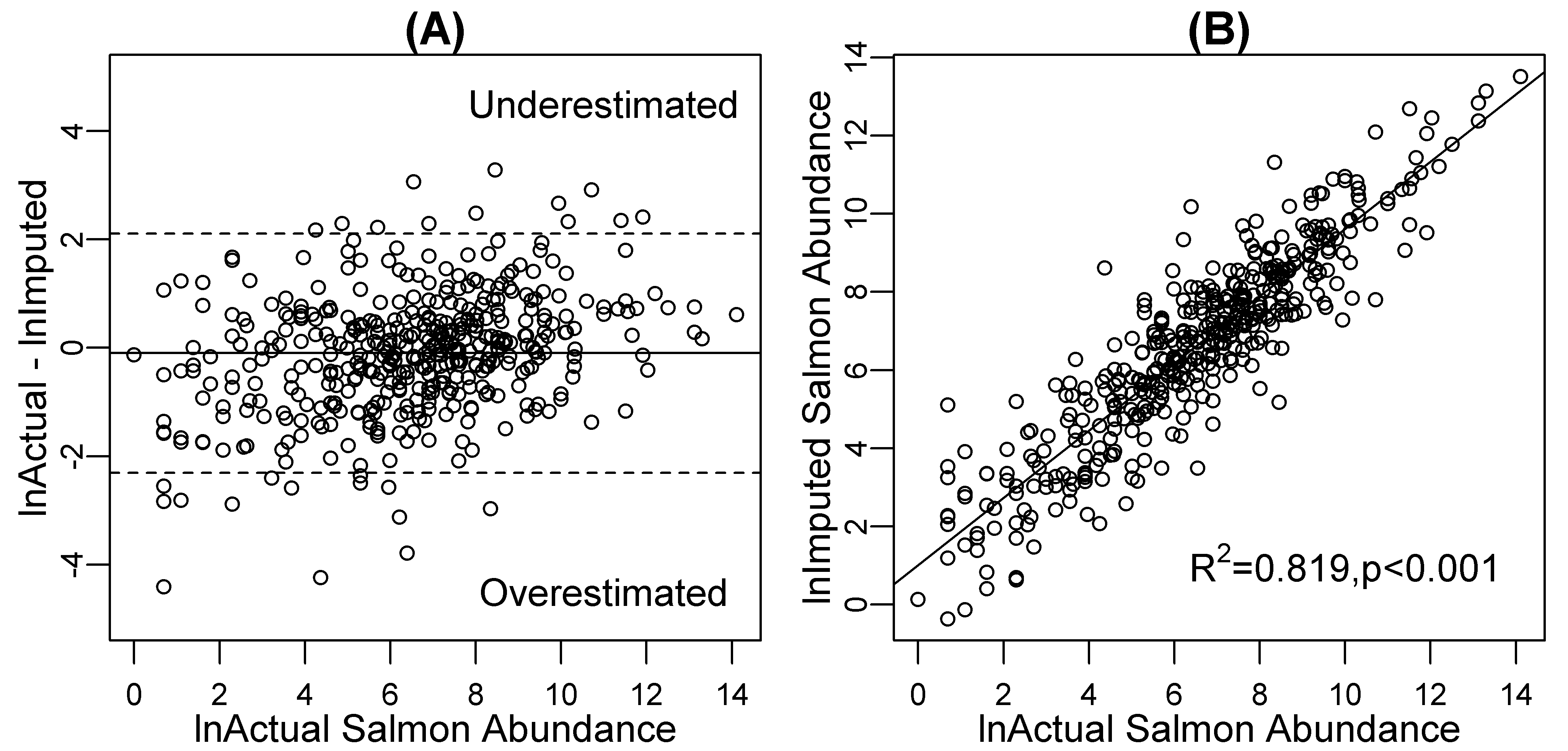


Figure S2. Correction factor applied to salmon biomass imputations for each species and watershed based on the number of missing data points in the last 15 years.

The correction factor was fully determined by 1) the threshold number of missing values after which a correction was applied (N_c_) and 2) the maximum magnitude of the correction (M_c_). An N_c_=15/8 and M_c_=0.4 adequately reduced overestimation of species-watershed combinations with low salmon abundance. Accordingly, we averaged available (non-missing) counts over the past 15 years and multiplied by this correction factor when computing the relative contributions of a watershed to species counts in a fisheries management area. We excluded watershed-species combinations with more than 10 missing values.


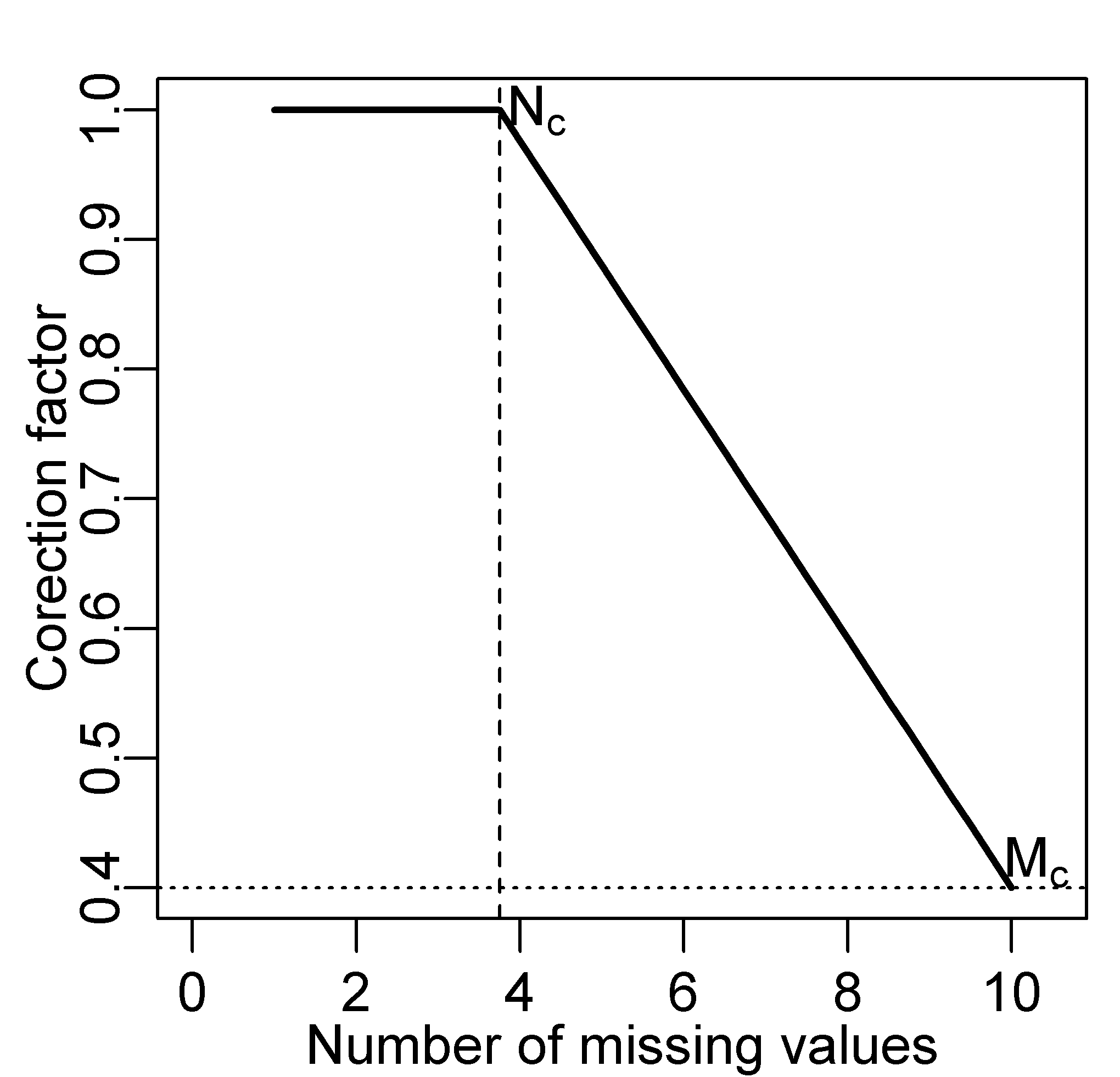


*Salmon biomass calculations*

To calculate salmon biomass available to each bear, we placed a buffer around each bear detection location based on estimated home range sizes for grizzly and black bears in coastal British Columbia. Because bears could have been detected anywhere in their home range, we generated a regular grid of 40 candidate buffers for each bear, with each buffer containing the bear’s detection location. Initially, we generated three sets of buffers for each bear, corresponding to the sex-specific minimum, mean and maximum home ranges reported from previous studies (Table S2; Machutch*on et a*l., 1993; H. Davis, unpublished data). The salmon biomasses calculated for the three buffer sizes were correlated with the exception of only one or two individuals per species (Fig. S3); we chose the maximum buffer size for further calculations because all home ranges encompassed spawning salmon streams as well as most location points for bears detected on multiple occasions. Moreover, a larger home range would be expected for bears inhabiting hyper-maritime ecosystems—which typically have lower productivity—than the maritime or sub-maritime ecosystems where these other studies were conducted (T. Hamilton, personal communication).

We then calculated the total 10-year average salmon biomass in each candidate buffer, summing across all streams in the buffer. We considered the buffer with the most salmon biomass to reflect best the salmon available to that bear. Additionally, we then used that buffer to calculate salmon biomass in the year of hair growth and the year prior to hair growth. For bears detected at multiple locations within the same year, we selected the location (and associated buffer) with the highest salmon biomass.

We assessed the adequacy of our home range estimates using bears detected at different locations or during different capture periods within or among years. In this analysis, we included bears without an adequate amount of hair for hormonal assays. Of 24 male black bears detected multiple times during the study, 20 (83%) had the same or similar buffers and salmon biomass estimates (median CV for salmon biomass estimates=0, range: 0-12%). The remaining 3 bears were detected in similar areas but had different biomass estimates because buffers were placed in different locations based on the proximity of a salmon spawning stream. One black bear moved a long distance (at least 75 km) within a 10-day hair collection period. In these four cases, we selected the buffer with the highest amount of salmon available, assuming that the bears maximize their access to this fitness-enhancing resource during the fall salmon season. Of 12 grizzly bears detected more than once, 7 had similar home ranges and salmon biomass estimates (CVs all <5%), 4 were detected in similar locations but had different biomass estimates because of buffer placements relative to salmon streams, and 1 had moved a minimum of 128 km between years. Similarly, as for black bears in cases with different salmon biomass estimates, we selected the buffer with the maximum amount of salmon available.

Finally, we performed a principal components analysis to differentiate between the long-term effects of salmon availability—reflecting the number of bears in an area—and year-to-year variability in salmon availability—which would affect the nutritional and social contexts. These measures became our “watershed productivity” and “salmon availability” variables, respectively. We expected that hormone levels in hair might relate to salmon-mediated conditions in the year of hair growth or in the previous year’s salmon spawning season. In both grizzly and black bears, the first principle component (PC1) aligned with average salmon abundance (“watershed productivity”) and the second (PC2) with increasing same-year salmon abundance (“salmon availability”) and decreasing previous-year salmon abundance (Fig. S4).

**Table S2.** Buffer sizes (km^2^) placed around grizzly and black bear sampling locations to estimate salmon biomass in an individual’s estimated home range. All home ranges were based on 100% minimum convex polygons for radio-collared bears in coastal BC.

|  | n | Min | Mean | Max | Source |
| --- | --- | --- | --- | --- | --- |
| Male Grizzly | 4 | 57 | 130 | 220 | Khutzeymateen Valley, (Machutchon et al. 1993) |
| Female Grizzly | 13 | 22.5 | 51.8 | 115.5 |
| Male Black | 12 | 45 | 128.8 | 253 | Nimpkish Valley, Vancouver Island (H. Davis, unpublished data) |
| Female Black | 9 | 7.32 | 11.3 | 23 |

Figure S3. Comparison of salmon biomass in mean and maximum home range buffers for (A) grizzly and (B) black male bears.

Minimum buffer sizes are not presented because of too many buffers in which salmon were absent.


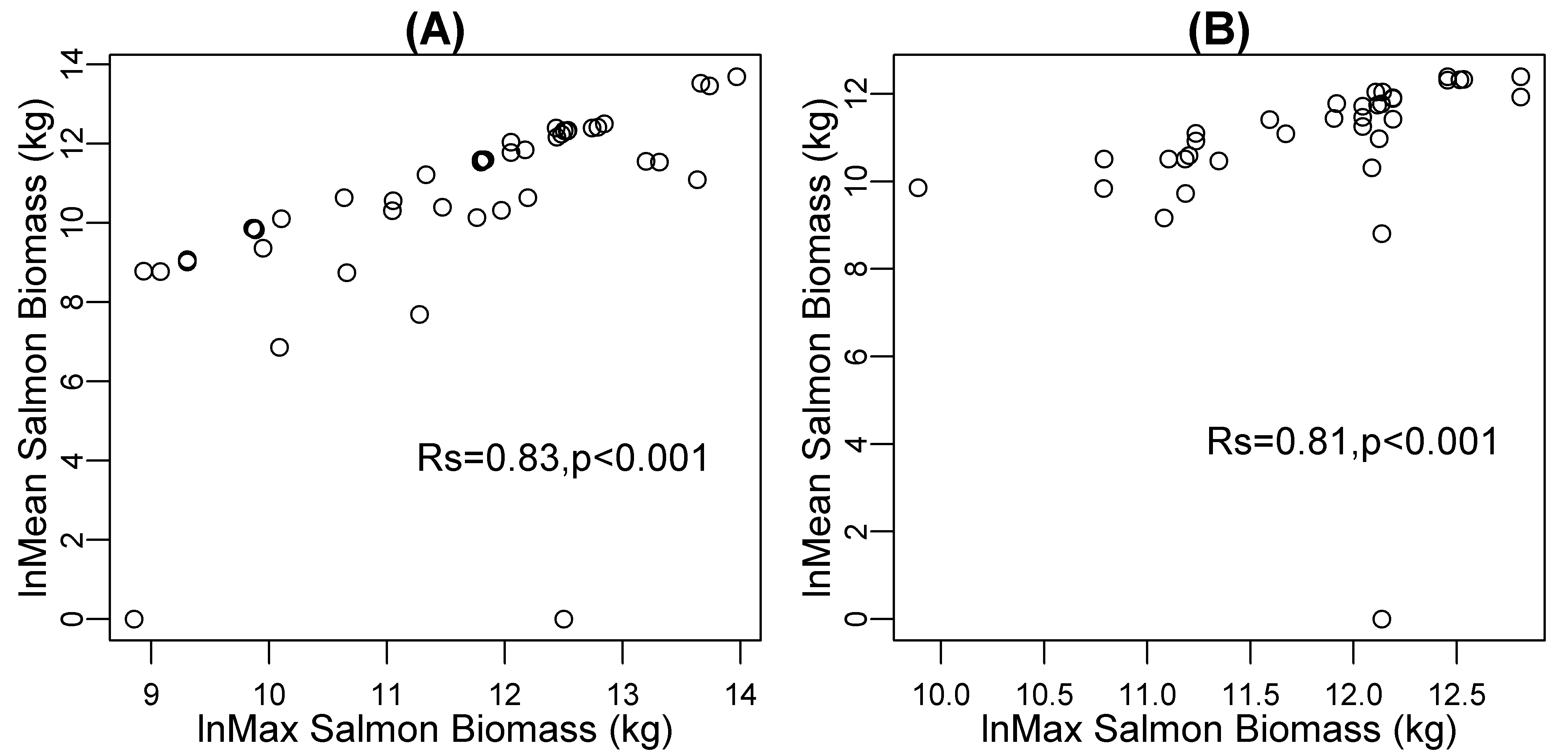


Figure S4. Biplot showing principal components for (A) grizzly bear and (B) black bear biomass variables. In both species, the first axis (PC1) aligned with average salmon biomass (“watershed productivity”) and the second (PC2) increased with same-year salmon biomass (“salmon availability”) and decreased with previous year salmon biomass.

Axes are set to the same scale. PC1 was multiplied by -1 to make interpretation more intuitive (i.e., increasing PC1 with increasing watershed productivity).


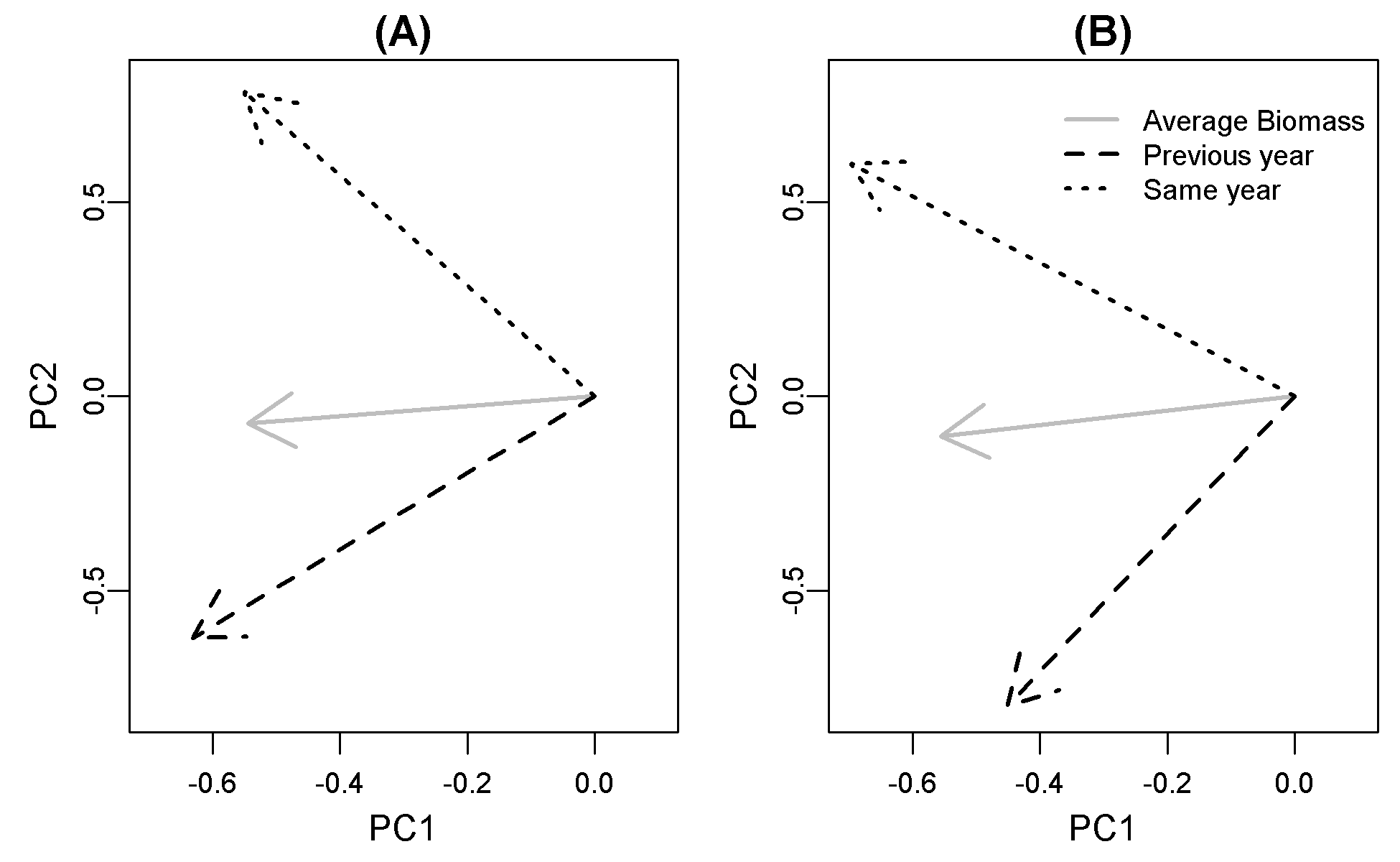


Table S3. Proportion of variation explained by each principal component (PC) and PC loadings for grizzly and black bear datasets.

|  | **Grizzly Bear n=54** | | | **Grizzly Bear n=27** | | | **Black Bear n=54** | | |
| --- | --- | --- | --- | --- | --- | --- | --- | --- | --- |
|  | **PC1** | **PC2** | **PC3** | **PC1** | **PC2** | **PC3** | **PC1** | **PC2** | **PC3** |
| Standard deviation | 2.30 | 1.15 | 0.42 | 1.54 | 1.34 | 0.33 | 1.20 | 0.89 | 0.25 |
| Proportion of Variance | 0.78 | 0.20 | 0.03 | 0.56 | 0.42 | 0.03 | 0.63 | 0.34 | 0.03 |
| Cumulative Proportion | 0.78 | 0.97 | 1.00 | 0.56 | 0.97 | 1.00 | 0.63 | 0.97 | 1.00 |
| Average Biomass, i.e.,  Watershed Productivity | -0.54 |  | 0.84 | -0.44 | -0.15 | 0.89 | -0.56 |  | 0.83 |
| Previous Year Biomass | -0.65 | -0.62 | -0.43 | -0.42 | -0.84 | -0.35 | -0.62 | -0.68 | -0.40 |
| Same Year Biomass, i.e., Salmon Availability | -0.54 | 0.78 | -0.32 | -0.80 | 0.52 | -0.31 | -0.54 | 0.74 | -0.40 |

*Model sets (Tables S4-S9) and model validations (Figs. S5, S6) for variables explaining cortisol and testosterone in hair from male grizzly and black bears*

Model sets were derived from specific hypotheses outlined in the Introduction and Methods sections (Main Manuscript). Model sets are slightly different for each response variable because we first competed variables within each category of variables (Environmental or Individual variables) and then competed the top models from each category, as described in the methods (Main Manuscript). We inspected residuals of top models against all variables not included in the model to ensure we were not missing important explanatory variables. Some of our model validations are shown in Figs. S5 and S6. Additional validation steps are described in the Methods (Main Manuscript)

Table S4. Candidate models for variables explaining variation in **cortisol** in hair of **male grizzly bears at the larger spatial scale (n=54**), sampled from grizzly bears from across coastal British Columbia between 2003 and 2011. Models were categorized (Cat.) as environmental variables (Env.), individual variables (Ind.), or both. Minimum and maximum variance inflation factors (VIF) for variables in each model are presented.

| **Cat.** | **Model** | **AICc** | **∆AICc** | **ω** | **Min VIF** | **Max VIF** |
| --- | --- | --- | --- | --- | --- | --- |
| Ind. | Salmon Consumption | 153.8 | 0.0 | 0.28 | 0.0 | 0.0 |
| Both | Salmon Consumption + Watershed Productivity | 154.3 | 0.5 | 0.21 | 1.0 | 1.0 |
| Ind. | Testosterone + Salmon Consumption | 155.9 | 2.1 | 0.10 | 1.0 | 1.0 |
| Both | Salmon Consumption **×** Watershed Productivity | 156.0 | 2.3 | 0.09 | 1.0 | 1.0 |
| Env. | Watershed Productivity | 156.1 | 2.4 | 0.08 | 0.0 | 0.0 |
| Ind. | Testosterone **×** Salmon Consumption | 156.3 | 2.5 | 0.08 | 1.0 | 1.1 |
|  | **Null Model** | **156.5** | **2.7** | **0.07** | **0.0** | **0.0** |
| Ind. | Testosterone | 158.1 | 4.4 | 0.03 | 0.0 | 0.0 |
| Env. | Watershed Productivity + Salmon Availability | 158.2 | 4.5 | 0.03 | 1.0 | 1.0 |
| Env. | Salmon Availability | 158.5 | 4.7 | 0.03 | 0.0 | 0.0 |
| Env. | Watershed Productivity **×** Salmon Availability | 160.6 | 6.9 | 0.01 | 1.0 | 1.0 |

Table S5. Candidate models for variables explaining variation in **testosterone** in hair of **male grizzly bears at the larger spatial scale (n=54**), sampled from grizzly bears from across coastal British Columbia between 2003 and 2011. Models were categorized (Cat.) as environmental variables (Env.), individual variables (Ind.), or both. Minimum and maximum variance inflation factors (VIF) for variables in the models are presented. Interaction terms are abbreviated (Watershed Productivity = WP, Salmon Availability = SA, and Salmon Consumption = SC).

| **Cat.** | **Model** | **AICc** | **∆AICc** | **ω** | **Min VIF** | **Max VIF** |
| --- | --- | --- | --- | --- | --- | --- |
| Both | Salmon Consumption + Watershed Productivity + Salmon Availability + WP × SA + SC × SA | 152.4 | 0.0 | 0.26 | 1.0 | 1.1 |
| Env. | Watershed Productivity × Salmon Availability | 153.7 | 1.3 | 0.13 | 1.0 | 1.0 |
| Env. | Salmon Availability | 154.7 | 2.3 | 0.08 | 0.0 | 0.0 |
| Both | Cortisol + Watershed Productivity + Salmon Availability + WP × SA | 154.8 | 2.4 | 0.08 | 1.0 | 1.1 |
| Both | Salmon Consumption + Watershed Productivity + Salmon Availability + WP × SA | 155.2 | 2.8 | 0.06 | 1.0 | 1.1 |
| Both | Salmon Consumption + Watershed Productivity + Salmon Availability + WP × SA + SC × WP | 155.4 | 3.0 | 0.06 | 1.0 | 1.1 |
| Both | Cortisol + Watershed Productivity + Salmon Availability + WP × SA + Cortisol × SA | 155.6 | 3.2 | 0.05 | 1.0 | 1.1 |
| Both | Cortisol + Salmon Consumption + Watershed Productivity + Salmon Availability + WP × SA + SC × SA + Cortisol × SA | 156.0 | 3.6 | 0.04 | 1.0 | 1.2 |
| Env. | Watershed Productivity + Salmon Availability | 156.2 | 3.8 | 0.04 | 1.0 | 1.0 |
|  | **Null Model** | **156.5** | **4.1** | **0.03** | **0.0** | **0.0** |
| Both | Cortisol + Watershed Productivity + Salmon Availability + WP × SA + Cortisol × WP | 156.7 | 4.3 | 0.03 | 1.0 | 1.1 |
| Both | Cortisol + Salmon Consumption + Watershed Productivity + Salmon Availability + Watershed WP × SA | 156.9 | 4.5 | 0.03 | 1.0 | 1.2 |
| Both | Cortisol + Salmon Consumption + Watershed Productivity + Salmon Availability + WP × SA + SC × SA + Cortisol × WP | 157.1 | 4.7 | 0.02 | 1.1 | 1.2 |
| Ind. | Salmon Consumption | 157.8 | 5.4 | 0.02 | 0.0 | 0.0 |
| Env. | Watershed Productivity | 158.0 | 5.6 | 0.02 | 0.0 | 0.0 |
| Ind. | Cortisol | 158.1 | 5.8 | 0.01 | 0.0 | 0.0 |
| Both | Cortisol + Salmon Consumption + Watershed Productivity + Salmon Availability + WP × SA + SC × WP + Cortisol × SA | 158.4 | 6.0 | 0.01 | 1.0 | 1.2 |
| Both | Cortisol + Salmon Consumption + Watershed Productivity + Salmon Availability + WP × SA + SC × WP + Cortisol × WP | 159.5 | 7.1 | 0.01 | 1.1 | 1.2 |
| Ind. | Cortisol × Salmon Consumption | 159.8 | 7.4 | 0.01 | 1.1 | 1.2 |
| Ind. | Cortisol + Salmon Consumption | 159.9 | 7.5 | 0.01 | 1.1 | 1.1 |

Table S6. Candidate models for variables explaining variation in **cortisol** in hair of **male grizzly bears at the smaller spatial scale (n=27**), where samples were collected from grizzly bears at hair snagging stations on the central coast of British Columbia between 2009 and 2011. Models were categorized (Cat.) as environmental variables (Env.), individual variables (Ind.), or both. Minimum and maximum variance inflation factors (VIF) for variables are presented.

| **Cat.** | **Model** | **AICc** | **∆AICc** | **ω** | **Min VIF** | **Max VIF** |
| --- | --- | --- | --- | --- | --- | --- |
| Both | Salmon Consumption + Testosterone + Year + Black Bear Density | 65.8 | 0.0 | 0.79 | 1.0 | 1.3 |
| Both | Salmon Consumption + Year + Black Bear Density | 69.9 | 4.1 | 0.10 | 1.0 | 1.3 |
| Both | Salmon Consumption + Testosterone + Year | 72.6 | 6.8 | 0.03 | 1.1 | 1.3 |
| Both | Salmon Consumption + Year | 73.2 | 7.4 | 0.02 | 1.1 | 1.3 |
| Env. | Year + Black Bear Density | 73.8 | 8.1 | 0.01 | 1.0 | 1.0 |
| Env. | Year | 74.4 | 8.6 | 0.01 | 0.0 | 0.0 |
| Env. | Year + Grizzly Bear Density + Black Bear Density | 75.7 | 9.9 | 0.01 | 1.0 | 1.0 |
| Env. | Year + Grizzly Bear Density | 75.9 | 10.1 | 0.01 | 1.0 | 1.0 |
| Ind. | Salmon Consumption | 75.9 | 10.1 | 0.00 | 0.0 | 0.0 |
| Env. | Year + Salmon Availability + Black Bear Density | 77.1 | 11.3 | 0.00 | 1.1 | 2.7 |
| Ind. | Testosterone + Salmon Consumption | 77.2 | 11.4 | 0.00 | 1.0 | 1.0 |
| Env. | Year + Salmon Availability | 77.2 | 11.4 | 0.00 | 1.6 | 2.5 |
| Env. | Year + Salmon Availability + Grizzly Bear Density | 78.8 | 13.0 | 0.00 | 1.1 | 2.5 |
| Env. | Grizzly Bear Density | 79.1 | 13.3 | 0.00 | 0.0 | 0.0 |
| Env. | Year + Salmon Availability + Grizzly Bear Density + Black Bear Density | 79.4 | 13.6 | 0.00 | 1.1 | 2.8 |
| Env. | Salmon Availability + Black Bear Density | 80.0 | 14.2 | 0.00 | 1.1 | 1.1 |
|  | **Null Model** | **80.1** | **14.3** | **0.00** | **0.0** | **0.0** |
| Ind. | Testosterone | 80.1 | 14.3 | 0.00 | 0.0 | 0.0 |
| Ind. | Testosterone **×** Salmon Consumption | 80.2 | 14.4 | 0.00 | 1.1 | 1.2 |
| Env. | Salmon Availability + Grizzly Bear Density + Black Bear Density | 80.2 | 14.4 | 0.00 | 1.0 | 1.1 |
| Env. | Salmon Availability + Grizzly Bear Density | 80.5 | 14.7 | 0.00 | 1.0 | 1.0 |
| Env. | Salmon Availability | 80.7 | 14.9 | 0.00 | 0.0 | 0.0 |
| Env. | Watershed Productivity | 82.2 | 16.4 | 0.00 | 0.0 | 0.0 |
| Env. | Watershed Productivity + Salmon Availability | 83.0 | 17.2 | 0.00 | 1.0 | 1.0 |
| Env. | Watershed Productivity **×** Salmon Availability | 86.1 | 20.3 | 0.00 | 1.0 | 1.1 |

Table S7. Candidate models for variables explaining variation in **testosterone** in hair of **male grizzly bears at the smaller spatial scale (n=27)**, where samples were collected from grizzly bears at hair snagging stations on the central coast of British Columbia between 2009 and 2011. Models were categorized (Cat.) as environmental variables (Env.), individual variables (Ind.), or both. Minimum and maximum variance inflation factors (VIF) for variables are presented.

| **Cat.** | **Model** | **AICc** | **∆AICc** | **ω** | **Min VIF** | **Max VIF** |
| --- | --- | --- | --- | --- | --- | --- |
| Both | Salmon Availability **×** Cortisol | 74.9 | 0.0 | 0.62 | 1.0 | 1.1 |
| Both | Salmon Availability **+** Cortisol | 78.5 | 3.6 | 0.10 | 1.1 | 1.1 |
|  | **Null Model** | **80.1** | **5.2** | **0.05** | **0.0** | **0.0** |
| Ind. | Cortisol | 80.1 | 5.2 | 0.05 | 0.0 | 0.0 |
| Env. | Salmon Availability | 80.5 | 5.6 | 0.04 | 0.0 | 0.0 |
| Env. | Grizzly Bear Density | 81.4 | 6.4 | 0.03 | 0.0 | 0.0 |
| Ind. | Salmon Consumption | 81.5 | 6.6 | 0.02 | 0.0 | 0.0 |
| Env. | Watershed Productivity | 82.1 | 7.2 | 0.02 | 0.0 | 0.0 |
| Env. | Salmon Availability + Black Bear Density | 82.2 | 7.3 | 0.02 | 1.1 | 1.1 |
| Env. | Black Bear Density | 82.3 | 7.4 | 0.01 | 0.0 | 0.0 |
| Env. | Salmon Availability + Grizzly Bear Density | 82.3 | 7.4 | 0.02 | 1.0 | 1.0 |
| Env. | Year | 82.4 | 7.4 | 0.02 | 0.0 | 0.0 |
| Env. | Watershed Productivity + Salmon Availability | 82.7 | 7.7 | 0.01 | 1.0 | 1.0 |
| Ind. | Cortisol + Salmon Consumption | 82.8 | 7.8 | 0.01 | 1.3 | 1.3 |
| Env. | Year + Grizzly Bear Density | 83.3 | 8.3 | 0.01 | 1.0 | 1.0 |
| Env. | Salmon Availability + Black Bear Density + Grizzly Bear Density | 84.4 | 9.5 | 0.01 | 1.0 | 1.1 |
| Env. | Year + Black Bear Density | 84.8 | 9.8 | 0.00 | 1.0 | 1.0 |
| Env. | Watershed Productivity **×** Salmon Availability | 84.8 | 9.9 | 0.00 | 1.0 | 1.1 |
| Env. | Year + Salmon Availability | 84.8 | 9.9 | 0.00 | 1.6 | 2.5 |
| Ind. | Salmon Consumption **×** Cortisol | 85.3 | 10.3 | 0.00 | 1.1 | 1.5 |
| Env. | Year + Grizzly Bear Density + Black Bear Density | 86.0 | 11.1 | 0.00 | 1.0 | 1.0 |
| Env. | Salmon Availability + Year + Grizzly Bear Density | 86.3 | 11.3 | 0.00 | 1.1 | 2.5 |
| Env. | Year + Salmon Availability + Black Bear Density | 86.8 | 11.8 | 0.00 | 1.1 | 2.7 |
| Env. | Year + Salmon Availability + Grizzly Bear Density + Black Bear Density | 88.8 | 13.9 | 0.00 | 1.1 | 2.8 |

Table S8. Candidate models explaining variation in **cortisol** in hair of **male black bears (n=59)**, sampled at hair snag stations on the central coast of British Columbia from 2009-2011. Models were categorized (Cat.) as environmental variables (Env.), individual variables (Ind.), or both. Minimum and maximum variance inflation factors (VIFs) for variables in each model are presented.

| **Cat.** | **Model** | **AICc** | **∆AICc** | **ω** | **Min VIF** | **Max VIF** |
| --- | --- | --- | --- | --- | --- | --- |
| Both | Salmon Consumption + Testosterone + Year + Salmon Availability | 139.3 | 0.0 | 0.24 | 1.1 | 2.1 |
| Both | Salmon Consumption + Testosterone + Year + Salmon Availability + Black Bear Density | 139.8 | 0.5 | 0.19 | 1.0 | 2.1 |
| Both | Salmon Consumption + Testosterone + Year + Salmon Availability + Salmon Availability **×** Salmon Consumption | 141.0 | 1.7 | 0.10 | 0.0 | 0.0 |
| Env. | Salmon Availability + Year + Black Bear Density | 141.4 | 2.1 | 0.09 | 1.0 | 2.0 |
| Both | Salmon Consumption + Testosterone + Year + Salmon Availability + Salmon Availability **×** Testosterone | 141.7 | 2.4 | 0.07 | 0.0 | 0.0 |
| Env. | Salmon Availability + Year + Grizzly Bear Density + Black Bear Density | 142.3 | 2.9 | 0.06 | 1.0 | 2.0 |
| Env. | Salmon Availability + Year | 142.9 | 3.6 | 0.04 | 1.4 | 2.0 |
| Both | Salmon Consumption + Testosterone + Year + Salmon Availability + Salmon Availability × Testosterone + Salmon Availability × Salmon Consumption | 143.3 | 4.0 | 0.03 | 0.0 | 0.0 |
| Env. | Year + Black Bear Density | 144.0 | 4.7 | 0.02 | 1.0 | 1.0 |
| Env. | Salmon Availability + Year + Grizzly Bear Density | 144.5 | 5.2 | 0.02 | 1.0 | 2.0 |
| Env. | Year + Grizzly Bear Density + Black Bear Density | 145.4 | 6.1 | 0.01 | 1.0 | 1.0 |
| Env. | Year | 147.1 | 7.7 | 0.01 | 0.0 | 0.0 |
| Env. | Year + Grizzly Bear Density | 149.1 | 9.8 | 0.00 | 1.0 | 1.0 |
| Env. | Salmon Availability + Black Bear Density | 162.0 | 22.7 | 0.00 | 1.0 | 1.0 |
| Env. | Salmon Availability + Grizzly Bear Density + Black Bear Density | 163.5 | 24.2 | 0.00 | 1.0 | 1.0 |
| Ind. | Salmon Consumption + Testosterone | 165.1 | 25.8 | 0.00 | 1.1 | 1.1 |
| Env. | Salmon Availability | 165.7 | 26.4 | 0.00 | 0.0 | 0.0 |
| Ind. | Salmon Consumption × Testosterone | 167.3 | 28.0 | 0.00 | 1.0 | 1.1 |
| Env. | Watershed Productivity + Salmon Availability | 167.5 | 28.2 | 0.00 | 1.0 | 1.0 |
| Env. | Salmon Availability + Grizzly Bear Density | 167.8 | 28.5 | 0.00 | 1.0 | 1.0 |
| Ind. | Testosterone | 169.0 | 29.6 | 0.00 | 0.0 | 0.0 |
| Env. | Watershed Productivity × Salmon Availability | 169.4 | 30.0 | 0.00 | 1.0 | 1.1 |
| Ind. | Salmon Consumption | 169.7 | 30.4 | 0.00 | 0.0 | 0.0 |
|  | **Null Model** | **170.6** | **31.3** | **0.00** | **0.0** | **0.0** |
| Env. | Watershed Productivity | 172.4 | 33.1 | 0.00 | 0.0 | 0.0 |

Table S9. Candidate models for variables explaining variation in **testosterone** in hair of **male black bears (n=59)**, collected at hair snagging stations on the central coast of British Columbia between 2009 and 2011. Models were categorized (Cat.) as environmental variables (Env.), individual variables (Ind.), or both. Minimum and maximum variance inflation factors (VIFs) for variables in each model are presented. Abbreviations are: WP=Watershed Productivity, SA=Salmon Availability, SC=Salmon Consumption.

| **Cat.** | **Model** | **AICc** | **∆AICc** | **ω** | **Min VIF** | **Max VIF** |
| --- | --- | --- | --- | --- | --- | --- |
| Both | Salmon Consumption + Cortisol + Watershed Productivity + Salmon Availability + SA × Cortisol | 154.5 | 0.0 | 0.35 | 1.1 | 1.2 |
| Both | Salmon Consumption + Cortisol + Watershed Productivity | 154.9 | 0.5 | 0.28 | 1.1 | 1.2 |
| Both | Salmon Consumption + Cortisol + WP + WP × SC | 156.6 | 2.2 | 0.12 | 1.1 | 1.4 |
| Both | Salmon Consumption + Cortisol + Watershed Productivity + Salmon Availability + SA × SC | 157.1 | 2.6 | 0.09 | 1.1 | 1.2 |
| Both | Salmon Consumption + Cortisol + Watershed Productivity + Watershed Productivity × Cortisol | 157.4 | 2.9 | 0.08 | 1.1 | 1.2 |
| Both | Salmon Consumption + Cortisol + Watershed Productivity + WP × SC + WP × Cortisol | 159.2 | 4.7 | 0.03 | 1.2 | 1.4 |
| Both | Salmon Consumption + Cortisol + WP + Salmon Availability + SA × SC + WP × Cortisol | 159.6 | 5.2 | 0.03 | 1.1 | 1.3 |
| Ind. | Salmon Consumption + Cortisol | 164.0 | 9.5 | 0.00 | 1.1 | 1.1 |
| Ind. | Salmon Consumption + Cortisol + SC × Cortisol | 166.1 | 11.6 | 0.00 | 1.0 | 1.1 |
| Env. | Watershed Productivity | 168.3 | 13.8 | 0.00 | 0.0 | 0.0 |
| Ind. | Salmon Consumption | 168.6 | 14.1 | 0.00 | 0.0 | 0.0 |
| Ind. | Cortisol | 169.0 | 14.5 | 0.00 | 0.0 | 0.0 |
| Env. | Watershed Productivity × Salmon Availability | 169.3 | 14.9 | 0.00 | 1.0 | 1.1 |
| Env. | Watershed Productivity + Salmon Availability | 169.4 | 15.0 | 0.00 | 1.0 | 1.0 |
| Env. | Watershed Productivity + Black Bear Density | 169.6 | 15.1 | 0.00 | 1.1 | 1.1 |
| Env. | Watershed Productivity + Grizzly Bear Density | 170.5 | 16.1 | 0.00 | 1.1 | 1.1 |
| **Env.** | **Null Model** | **170.6** | **16.2** | **0.00** | **0.0** | **0.0** |
| Env. | Salmon Availability | 171.8 | 17.3 | 0.00 | 0.0 | 0.0 |
| Env. | Watershed Productivity + Grizzly Bear Density + Black Bear Density | 172.0 | 17.5 | 0.00 | 1.1 | 1.2 |
| Env. | Watershed Productivity + Year | 172.9 | 18.4 | 0.00 | 1.0 | 1.0 |
| Env. | Watershed Productivity + Year + Black Density | 174.4 | 19.9 | 0.00 | 1.0 | 1.1 |
| Env. | Year + Black Bear Density | 174.9 | 20.4 | 0.00 | 1.0 | 1.0 |
| Env. | Year | 175.1 | 20.7 | 0.00 | 0.0 | 0.0 |
| Env. | Watershed Productivity + Year + Grizzly Density | 175.4 | 20.9 | 0.00 | 1.0 | 1.1 |
| Env. | Watershed Productivity + Year + Grizzly Bear Density + Black Bear Density | 177.0 | 22.5 | 0.00 | 1.0 | 1.1 |
| Env. | Year + Grizzly Bear Density + Black Bear Density | 177.0 | 22.5 | 0.00 | 1.0 | 1.0 |
| Env. | Year + Grizzly Bear Density | 177.4 | 23.0 | 0.00 | 1.0 | 1.0 |

Figure S5. Model validation for **cortisol** in grizzly and black bear hair. Subplots A, C, and E show histograms of residuals from the top model from each model set. The p-value is from a Kolmogorov-Smirnov normality test of the residuals. Subplots B, D, and E show plots of actual versus predicted values from each model. Additional validations are described in the methods.


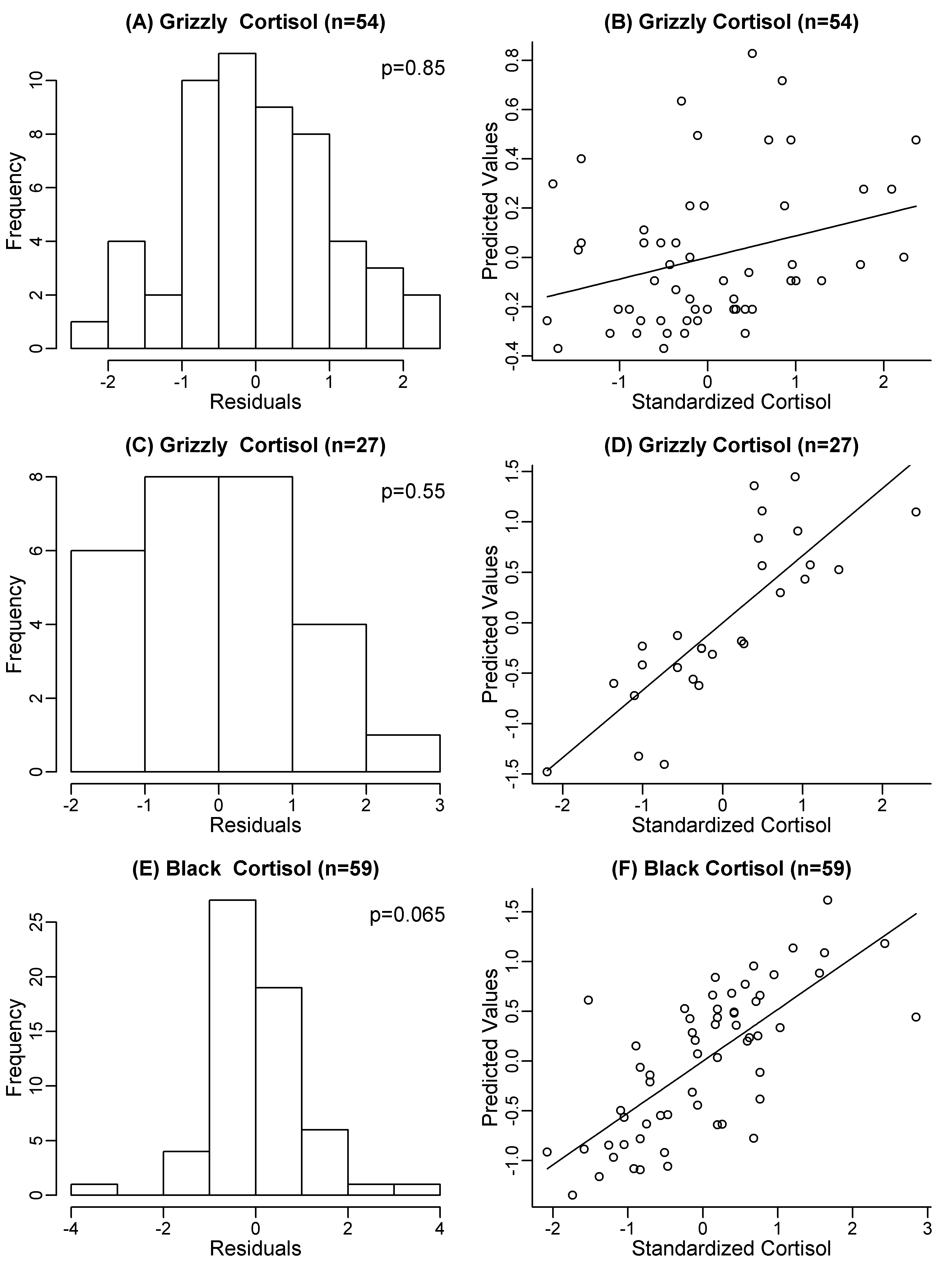


Figure S6. Model validations for **testosterone** in grizzly and black bear hair. Subplots A, C, and E show histograms of residuals from the top model from each model set. The p-value is from a Kolmogorov-Smirnov normality test of the residuals. Subplots B, D, and E show plots of actual versus predicted values from each model. Additional validations are described in the methods.


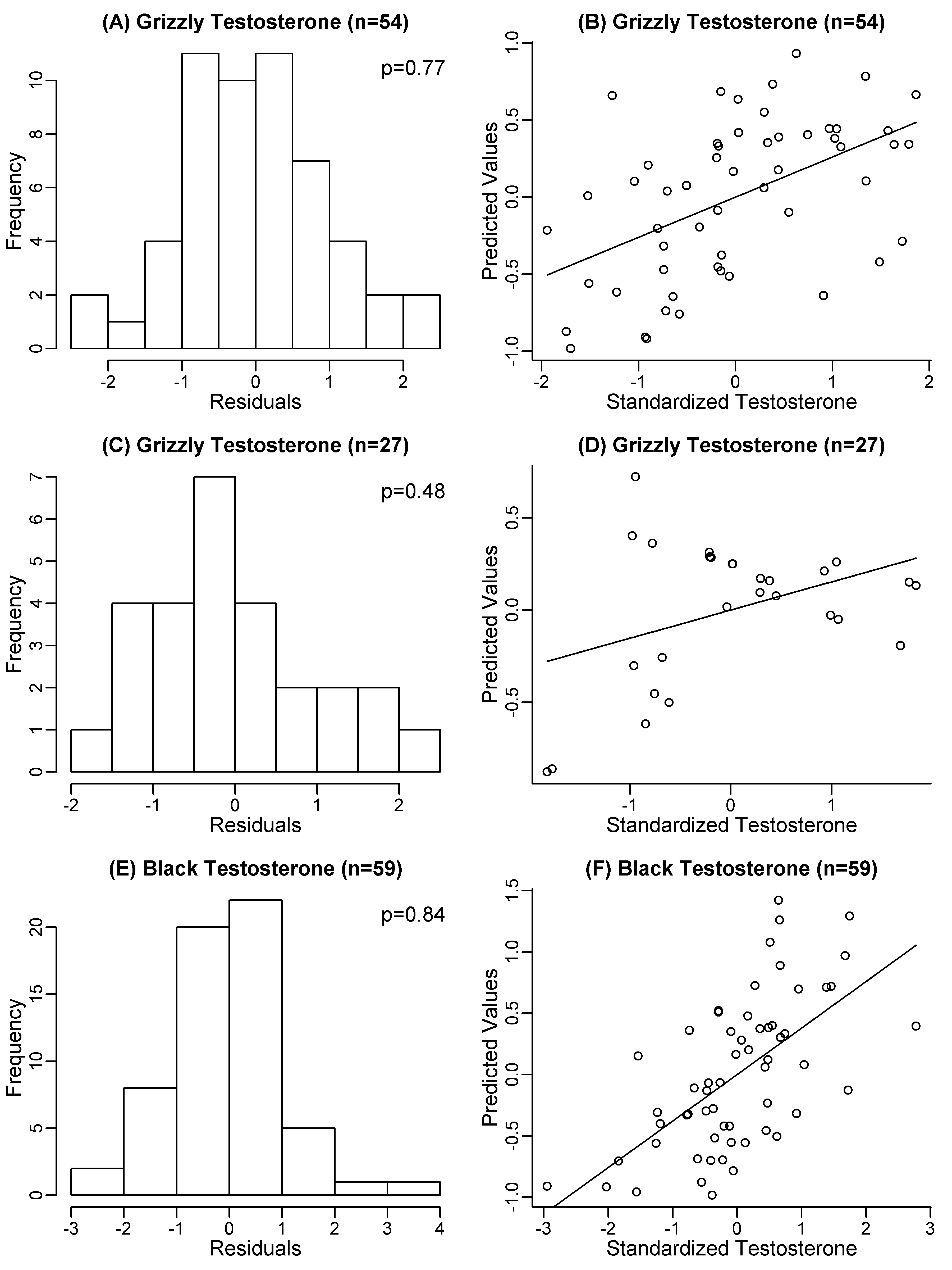

Supplement: Supplementary Data [file supp_cou010_cou010supp.doc]
